# Impact of Phylogenetic Method Choice on Indel Analyses in HIV-1 Subtype B

**DOI:** 10.1093/gbe/evaf119

**Published:** 2025-06-12

**Authors:** Mickaël Seppey, Clara Iglhaut, Manuel Gil, Maria Anisimova

**Affiliations:** Institute of Computational Life Science, Zürich University of Applied Sciences, Wädenswil, Switzerland; Swiss Institute of Bioinformatics, Lausanne, Switzerland; Institute of Computational Life Science, Zürich University of Applied Sciences, Wädenswil, Switzerland; Swiss Institute of Bioinformatics, Lausanne, Switzerland; Faculty of Mathematics and Science, University of Zurich, Zürich, Switzerland; Institute of Computational Life Science, Zürich University of Applied Sciences, Wädenswil, Switzerland; Swiss Institute of Bioinformatics, Lausanne, Switzerland; Institute of Computational Life Science, Zürich University of Applied Sciences, Wädenswil, Switzerland; Swiss Institute of Bioinformatics, Lausanne, Switzerland

**Keywords:** indel detection, phylogenetic analysis, multiple sequence alignment, ancestral sequence reconstruction, HIV-1

## Abstract

Insertions and deletions (indels) play a critical role in the evolutionary dynamics of genomes, yet their accurate detection and interpretation in phylogenetic studies remain challenging. Our study investigates the influence of different multiple sequence alignment (MSA) and ancestral sequence reconstruction (ASR) tools on indel pattern reconstruction, focusing on HIV-1 subtype B. We aim to understand how methodological choices affect the detection of indels, thereby emphasizing the importance of selecting appropriate tools for evolutionary analyses to improve phylogenetic accuracy. We conducted a comparative analysis using five MSA tools (MAFFT, ${\text{PRANK}}_{+F}$, IndelMaP, ProPIP, and Historian) and five ASR tools (GRASP, FastML, IndelMaP, ARPIP, and Historian). By examining inferred indel events across all tool combinations, we evaluated their rates, lengths, and positions within the genome, specifically analyzing the *env* gene and its V1 variable loop. Even though each method tested was able to reconstruct known variable regions in the *env* gene, our results highlight that the choice of MSA tool significantly impacts indel conservation and interpretation, more so than the choice of ASR tool. This finding underscores the necessity of context-specific MSA tool selection in phylogenetic studies and provides crucial insights for improving the accuracy of indel detection and evolutionary inferences in phylogenetic studies of HIV-1 and other genomes.

Significance Insertions and deletions (indels) are essential for understanding genome evolution, but accurately detecting and interpreting them remains difficult in phylogenetic studies. This study reveals that the choice of multiple sequence alignment (MSA) tools has a more significant effect on indel detection and conservation than the choice of ancestral sequence reconstruction tools. These findings underscore the importance of carefully selecting appropriate MSA tools in phylogenetic analyses, improving the accuracy of indel detection and evolutionary insights.

## Introduction

Genetic evolution is shaped by various factors, with insertion and deletion (indel) events playing a critical but often overlooked role. These genetic changes significantly contribute to genomic diversity and are important drivers of adaptive selection in different organisms (Wood et al. 2009; Miles et al. 2016; Zeng et al. 2018; Rogozin et al. 2024). In cancer genomics, indels serve as biomarkers with prognostic significance (Wu et al. 2019), and they offer insights into protein evolution and engineering (Studer et al. 2013; Savino et al. 2022). Indels, despite their significance, are often overlooked by phylogenetic methods. The overall pattern of indel events is usually represented as gaps in alignments of observed sequences, with gaps often treated as ambiguous characters or missing data, which may result in inaccuracies in subsequent analyses (Löytynoja and Goldman 2008). Reconstructing the history of individual indel events over time requires distinguishing insertions and deletions, whether using modeling or an algorithmic approach. Mathematically formulating the insertion–deletion process remains a significant challenge (Redelings et al. 2024). Unlike substitutions, indels often involve multiple sites, vary in length, and may overlap, making probabilistic descriptions complex. The computation of marginal likelihood under classical indel models, such as TKF91 (Thorne et al. 1991) and TKF92 (Thorne et al. 1992), is exponential in complexity due to the lack of site independence assumption. Nevertheless, several attempts with different approaches to handle indels have been made, leading to the development of various tools designed to better incorporate indel events in phylogenetic analyses.

MAFFT (Katoh et al. 2002), a widely used multiple sequence alignment (MSA) tool, works like most current progressive MSA methods, first aligning the sequences at the tips of the phylogeny and progressively building up towards the root. MAFFT enhances efficiency by employing Fast Fourier Transform to identify and prealign homologous segments prior to classical dynamic programming. While it is exceptionally fast, MAFFT like many MSA programs does not treat insertions and deletions as separate events, placing gaps based on the similarity of bordering residues, which can lead to over-alignment. Although a new option has been implemented to combat this issue, the approach does not distinguish between insertions and deletions (Katoh and Standley 2016). PRANK (Löytynoja and Goldman 2005) introduces a phylogeny-aware alignment method for MSA that treats insertions and deletions as distinct events. The algorithm uses outgroup information from the next alignment step to determine whether a length difference between observed sequences is due to an insertion or deletion. When gaps are placed, they are marked with a flag. In the subsequent alignment step, PRANK either confirms the gap or removes the flag, clarifying whether the event is an insertion or a deletion. This means gaps are marked as uncertain until the next step provides enough information to determine whether the event is an insertion or a deletion. ${\text{PRANK}}_{+F}$ (Löytynoja and Goldman 2008) is a modified version that uses flagged gap information to identify sites where the gap functions as a permanent insertion, ensuring they are not matched with a residue in subsequent progressive alignment steps. This approach prevents the erroneous grouping of independent indels by treating gaps as uncertain until clarified by the subsequent step, avoiding situations where overlapping deletions confirm embedded insertions. Used for ancestral sequence reconstruction (ASR) only, FastML was one of the first approaches that did not handle gaps as missing data. FastML addresses indels by encoding them as binary characters and using a maximum likelihood method to compute posterior probabilities for each indel site. It then infers the most likely character states at nongapped positions, combining indel and character reconstruction to provide posterior probabilities for each.

Although these approaches are indel-aware and allow insertions and deletions to be considered as distinct events, they use an evolutionary model only for substitutions and do not explicitly model indels. In contrast, ProPIP (Maiolo et al. 2021) and ARPIP (Jowkar et al. 2023), developed for MSA and ASR respectively, use an explicit mathematical model to account for indel evolution. They utilize the Poisson Indel Process (PIP; Bouchard-Côté and Jordan 2013), which treats insertions as Poisson events occurring throughout a phylogeny. Insertions are placed one residue at a time, evolving via a Markov process of substitutions and deletions along the phylogeny, with deletions modeled as an absorbing state. ProPIP uses PIP to progressively build an MSA by aligning the homology paths of the two children by full maximum likelihood. The inferred gap patterns are consistent with phylogenetic relationships but require cubic cost in the sequence length. ARPIP employs an empirical Bayesian approach with maximum likelihood estimates to infer the positions of insertion and deletion events on the tree according to the Poisson process. Ancestral states are reconstructed for subtrees defined by insertion points using an algorithm similar to that used by FastML, along with a modified version of Felsenstein’s recursion (Felsenstein 1981). However, both ProPIP and ARPIP are based on a single-residue indel model and may be inadequate for dealing with longer events.

While using evolutionary long indel models for MSA remains too costly, the necessity to analyze larger data sets encourages the development of faster tools. IndelMaP (Iglhaut et al. 2024), used for both MSA and ASR, is based on Dollo’s Law (Farris 1977), stating that a deleted residue cannot reappear, and extends Fitch’s algorithm (Fitch 1971). The progressive MSA method uses the same flagging algorithm as ${\text{PRANK}}_{+F}$ to separate insertion and deletion events. During ASR, the Dollo insertion point is first identified for each residue by placing it on the branch, leading to the most recent common ancestor of each leaf containing a residue at the site. The phylogeny is then split in two, and all character gaps appearing in the subtree defined by this Dollo insertion point are placed following a deletion, while all gaps appearing outside this subtree are treated as placeholders to signify the absence of a residue at that point in the tree. In addition to treating insertions and deletions as distinct evolutionary events, this method also allows longer indels to be taken into account and modeled using an affine gap scoring scheme. While operating column-by-column during ancestral sequence reconstruction, IndelMaP takes dependencies between gapped columns into account. Specifically, it distinguishes whether a gap is part of a longer insertion or deletion, ensuring that gaps within a continuous indel are treated differently from cases where a gap character in the previous column merely acts as a placeholder. Also based on parsimony, GRASP (Ross et al. 2022) is a different approach for ASR, using a partial order graph (POG) to model indels, a graphical structure in which vertices represent alignment columns and edges represent column order. This allows many possible gap patterns to be represented on the same graph, since a gap is depicted by an edge that skips one or more vertices. A parsimony score can then be computed for each path through the POG. This process, known as bidirectional edge parsimony, is carried out in both possible reading directions of the POG, enabling the identification of the path that is optimally parsimonious in both directions. A further advantage of this method is that it enables visualization of the ambiguity between all indel histories that present a close parsimony score and are, therefore, equally plausible given the MSA. GRASP is applicable only to protein sequences.

Modelling indels using a data structure similar to a POG, Historian (Holmes 2017) estimates alignments by considering the evolutionary history of both substitutions and indels. This tool generalizes Felsenstein’s pruning algorithm (Felsenstein 1981) and infers a subset of all possible histories where most of the probability mass is concentrated rather than relying on a single “best-guess” reconstruction. The resulting subset is generated through a stochastic traceback (Westesson et al. 2012). Notably, Historian can also be used for estimating ancestral sequences and evolutionary rates directly. By iterating through local alignments, Historian iteratively refines the input guide alignment provided by another tool.

In a previous study (Iglhaut et al. 2024), we compared most of these methods using simulated data to pinpoint the positions of indel events in the phylogenetic tree. However, the reliance on a molecular evolution model for simulation introduces bias, as models never fully reflect biological reality. This might also favor a tool due to the model used. Given that the inherent challenge of the field is the lack of ground truth, we chose to work here exclusively with real, nonsimulated data to overcome this issue. Our goal is to evaluate and contrast the different approaches on actual datasets, providing a more fair evaluation of these methods and exploring what insights can be gained from the data using these tools. We chose to apply them to an indel analysis of human immunodeficiency virus (HIV): Since its discovery over 40 years ago, it has left an undeniable mark on the infectious disease landscape and remains a global health challenge. HIV-1 is known to have an intricate and constantly evolving genetic diversity (Hemelaar et al. 2019), and that variability constitutes a substantial obstacle to the development of a globally effective vaccine (Gaschen et al. 2002). A better understanding of its underlying evolutionary mechanisms paves the way for more effective prevention strategies. Substitutions in the HIV genome have been studied in depth on the whole genome (Patiño-Galindo and González-Candelas 2017) and by comparing mutation rates between various genomic regions, in particular, the one coding for the envelope glycoprotein (Geller et al. 2015), but also the ones coding for Nef or Tat proteins (Poon et al. 2010; Ruiz et al. 2019; Williams et al. 2022).

For indels, on the other hand, fewer studies have been carried out, and insertion and deletion events are generally classified under the generic term indel or considered missing data. While the role of indels in the HIV genome and their impact on virus transmissibility, virulence, and treatment adaptation remain poorly understood due to them being hard to observe, some studies have already tried to shed some light on their pattern (Palmer and Poon 2019) and their function (Wood et al. 2009). The *env* gene, encoding the glycoprotein that interacts with antibodies, experiences strong selective pressure from the host immune system. This pressure likely drives its higher mutation rate, facilitating immune escape (Maphumulo and Gordon 2024). Wood and colleagues showed that indels in the *env* gene’s variable regions affect neutralization sensitivity and that inactivating indels (e.g. frameshifts) were evenly distributed across the *env* gene. In contrast nucleotide indels of lengths divisible by 3, or those compensated by another indel, were concentrated in the hypervariable regions V1, V2, V4, and V5. This concentration suggests selection pressure rather than an intrinsic mutational mechanism. Their study used intrapatient alignment to determine indel direction relative to the consensus sequence, presumed to be the transmitted strain. Palmer and colleagues on their side estimated indel rates from length differences between homologous sequences, considering their separation distance. This method reports indel events as binomial outcomes and does not account for aggregated events or multiple indels that cancel out sequence length variation.

Leveraging indel-aware methods could be a systematic way to study indels in the HIV-1 genome. Thus, we explored how the different methods and their underlying assumptions affect the outcome. We present here the results of our comparative method evaluation for both MSA and ASR with different strategies for indel handling. We used MAFFT, PRANK, IndelMaP, ProPIP, and Historian to generate MSAs, and then applied these alignments as input for ASR with GRASP, FastML, IndelMaP, ARPIP, and Historian, as part of a study of insertions and deletions in the nine HIV-1 subtype B genes. Here, we describe the differences and features of the 25 combinations tested, as well as the emerging patterns.

## Results

**Fig. 1.**
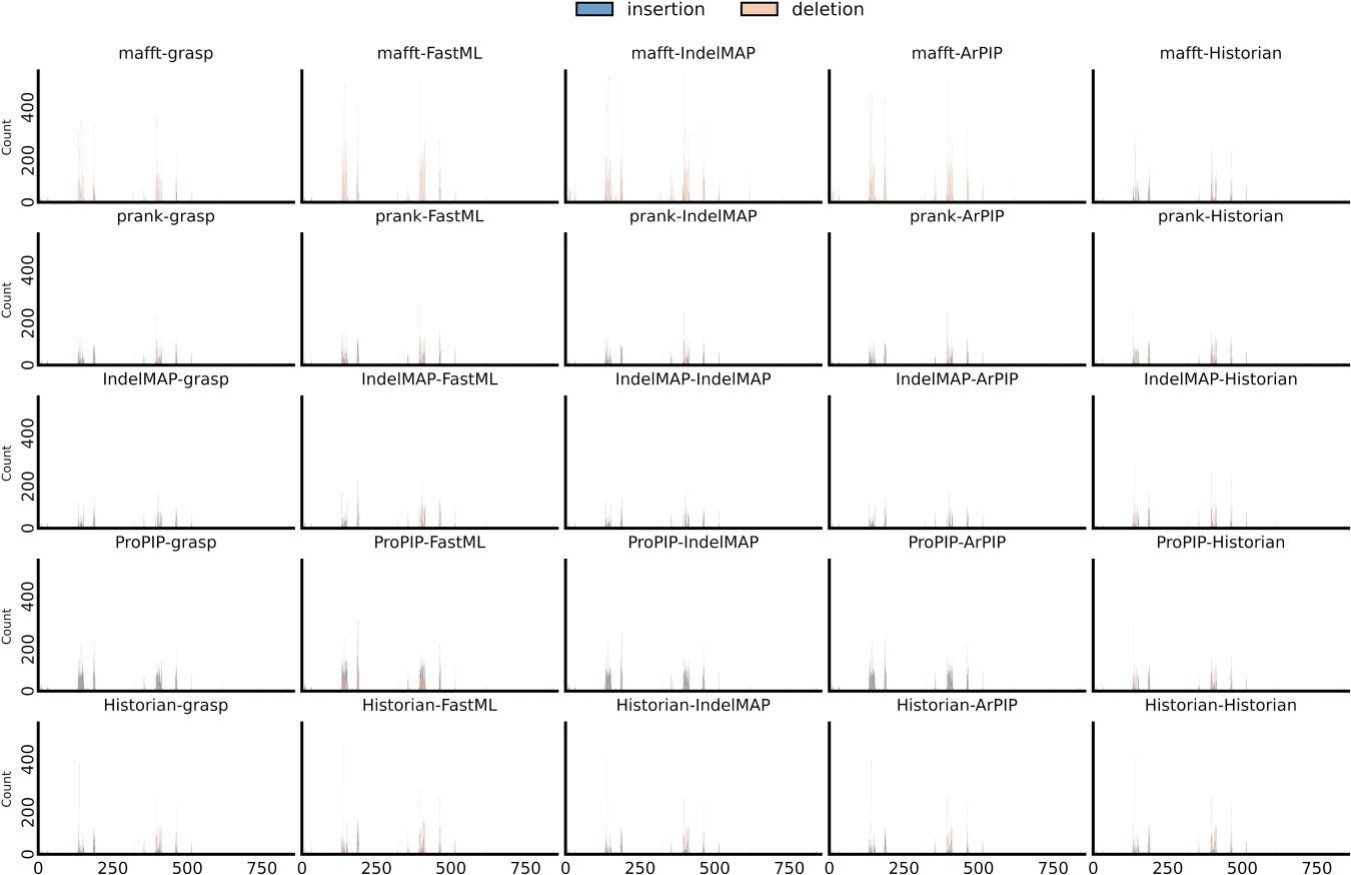
Insertion and deletion hotspots on the full *env* gene according to the various phylogenetic methods used. Abscissa shows the position of the event on the reference sequence K03455 and ordinate shows the count of events. Inserted sites, which inherently are not present in the reference sequence are mapped to half-integer positions. Note that Tools following Dollo’s law (i.e. IndelMaP) do not allow deleted residues to reappear, resulting in a maximal count of one insertion at integer positions.

For all nine HIV-1 subtype B genes, we reconstructed ancestral sequences using 25 different method combinations, involving five alignments (MAFFT, ${\text{PRANK}}_{+F}$, IndelMaP, ProPIP, Historian) and five ancestor reconstructions (GRASP, FastML, IndelMaP, ARPIP, Historian) based on the alignments. This strategy enables a comparative analysis of the different approaches and their impact on the resulting inferred indel pattern. For each region of interest and each combination, we extracted a list of inferred indel events specifying their length, their position in the phylogeny, and the position of the relevant residue relative to the K03455 reference sequence. This was done by traversing the phylogeny and comparing each alignment position with its parent sequence. We identified regions with the highest number of inferred indel events and show indel hotspots as bar plots for each combination (Fig. 1). For clarity reasons, here we only display the *env* gene, given that insertions and deletions are known to impact this region of the HIV genome. We further identified recognizable indel hotspots in the *gag* and *nef* regions (supplementary figs. S1 and S2, Supplementary Material online), as well as to a lesser extent in the *vpr* and *vpu* regions (supplementary figs. S7 and S8, Supplementary Material online). Other genes were found to be much less prone to indel events (supplementary figs. S3–S6, Supplementary Material online).

**Fig. 2.**
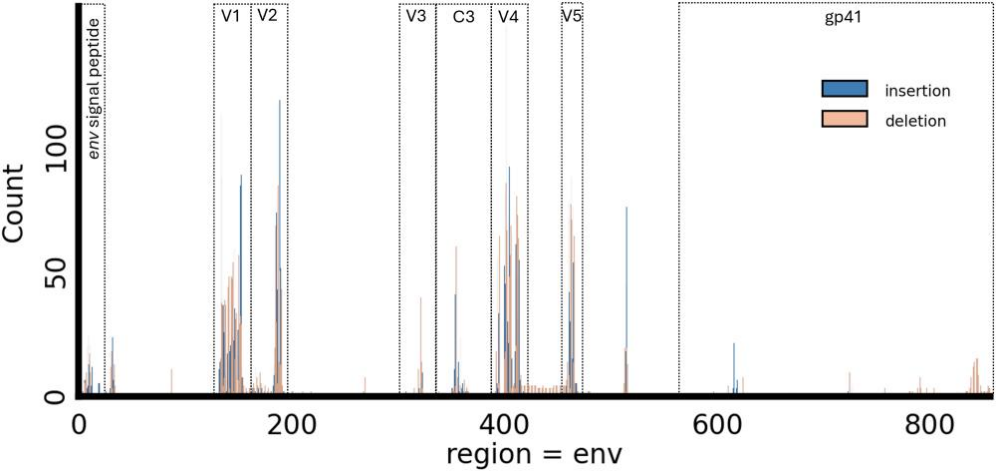
Insertion and deletion hotspots on the whole *env* gene according to the IndelMaP–IndelMaP method. The different parts of the gene (i.e. signal peptide, V1–V5 hypervariable loops, C3 conserved region and gp41) are highlighted. Inserted sites, which inherently are not present in the reference sequence are mapped to half-integer positions.

The chosen gene contains several regions of interest that have been extensively studied, though research on insertions and deletions within these regions is limited (Fig. 2). The first 30 amino acids correspond to the signal peptide. The following 481 form the glycoprotein gp120, separated into five conserved (C1–C5) and variable (V1–V5) regions. Finally, the last 345 amino acids compose the glycoprotein gp41.

Regions most prone to indels are placed similarly by all combinations of MSA and ASR. These are the hypervariable loop V1 extending from position 129 to 156, V2 extending from 157 to 196, the conserved region C3 between 332 and 382, the hypervariable loop V4 between 383 and 418, and V5 between 459 and 469. A hotspot is also observed between residues 5 and 15 in the *env* signal peptide region, as well as between 510 and 520, between 620 and 630, and finally between 830 and 855 in the gp41 protein coding region. Peak positions and profiles are very similar between the different ASRs used, but a noteworthy difference is observed between the MSA tools, with counts ranging for the largest peak from 150 for IndelMaP to 500 for MAFFT. These differences in inferred indel patterns become particularly pronounced when focusing on specific gene regions. Therefore, we have chosen to analyze distinct regions of the *env* gene (e.g. signal peptide, V1–V5, C1–C5, gp41) in greater detail, particularly concentrating on known hypervariable regions to better capture significant variations.

**Fig. 3.**
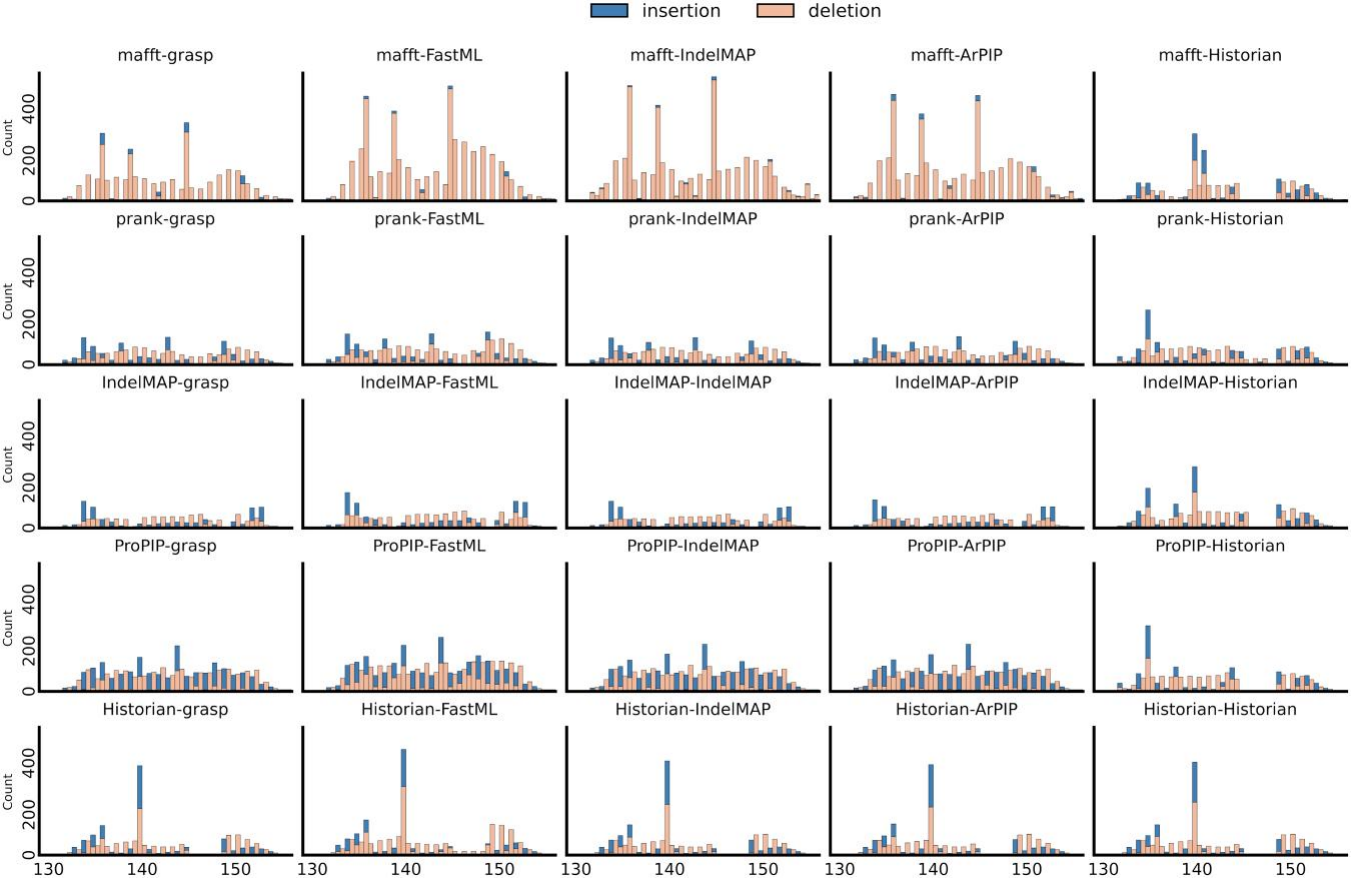
Insertion and deletion hotspots on the hypervariable loop V1 of *env* gene according to the various phylogenetic methods used. Abscissa shows the position of the event on the reference sequence K03455 and ordinate shows the count of events. Inserted sites, which inherently are not present in the reference sequence are mapped to half-integer positions.

At this scale, more obvious dissimilarities are apparent between the various combinations (Fig. 3). The indel patterns appear to be broadly preserved between the different reconstruction tools but varies between alignment tools. Historian seems to break out of this trend, although the patterns it infers using other alignments still slightly deviate from those obtained using its own alignment. The proportion between insertions and deletions shows a similar behavior, highly conserved between reconstruction tools while varying between alignments. This effect is particularly striking in the case of MAFFT, which shows a proportion of insertions close to zero compared to other alignments, except when reconstruction is carried out with Historian. Overall, the impact of alignment on the indel profile is substantially more pronounced than the choice of tool for ancestral sequence reconstruction. This observation was further demonstrated by computing the Wasserstein distances between the distributions of indels inferred by each method on the V1 hypervariable loop (supplementary fig. S9, Supplementary Material online). The mean Wasserstein distance for comparisons among different MSA tools was found to be 0.9685, while the mean distance for comparisons among different ASR tools was significantly lower, at 0.5185. The statistical significance of this difference was assessed using a Mann–Whitney *U* test, resulting in a U-statistic of 1,931.0 and a *P*-value of $2.72\times 10^{-6}$.

**Fig. 4.**
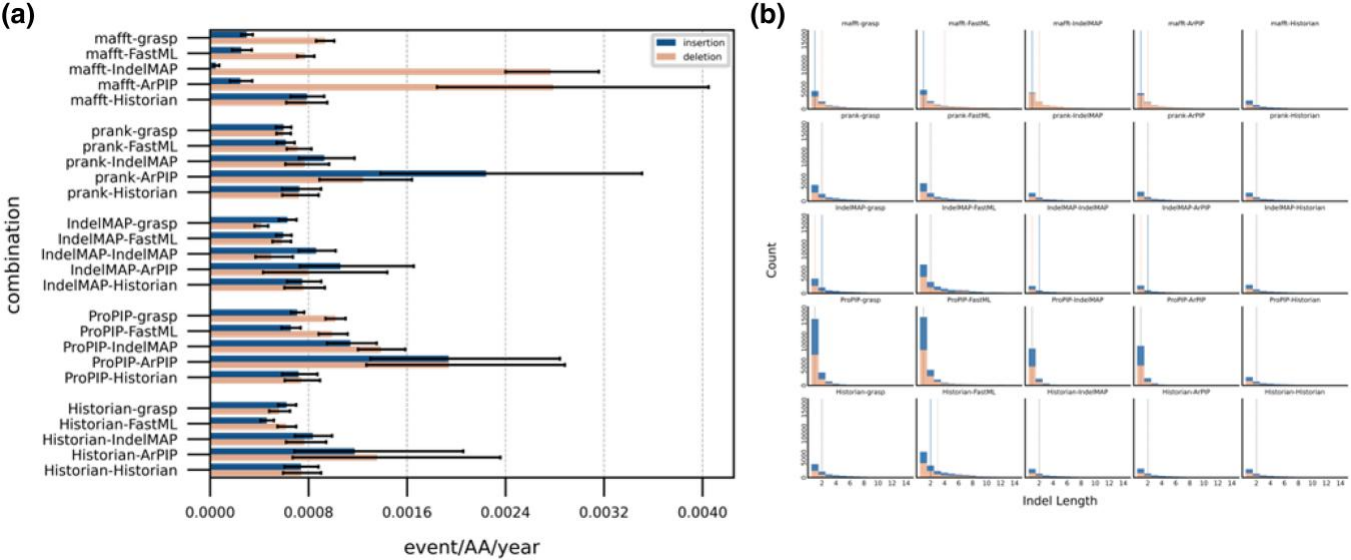
Indel rates (a) and indel lengths (b) comparison between methods for the full *env* gene.

We calculated the length of each indel by treating several successive residues inserted or deleted along the same branch as a single event. Given the length of each branch in the phylogenic trees and the count of events occurring along these branches, we could estimate the rates of insertions and deletions. Rates for the complete *env* gene, calculated as events per amino acid per year, are ranging for insertions from $4.83\times 10^{-5}$ (MAFFT-IndelMaP) to $2.24\times 10^{-3}$ (${\text{PRANK}}_{+F}$-ARPIP), and for deletions from $4.13\times 10^{-4}$ (IndelMaP-GRASP) to $2.79\times 10^{-3}$ (MAFFT-ARPIP) (Fig. 4). Deletion rates tend to be considerably higher than insertion rates whenever the MAFFT alignment is used, with an average of $1.24\times 10^{-3}$ and $1.43\times 10^{-4}$ for deletions and insertions, respectively. Other combinations show close rates between insertions and deletions. Regardless of the alignment used, we always observe higher rates with ARPIP and IndelMaP reconstructions compared to those obtained with FastML, GRASP, or Historian reconstructions. ARPIP shows the highest rates in each case. These findings also hold true for other HIV-1 subtype B genes analyzed, in particular the strong inclination of MAFFT to infer unbalanced deletion rates (supplementary figs. S11–S18, Supplementary Material online). We also found unusually high rates in *gag* and *vif* when ProPIP was used for alignment (supplementary figs. S11 and S16, Supplementary Material online). The results reported for other genes are consistent with those reported for *env*.

**Fig. 5.**
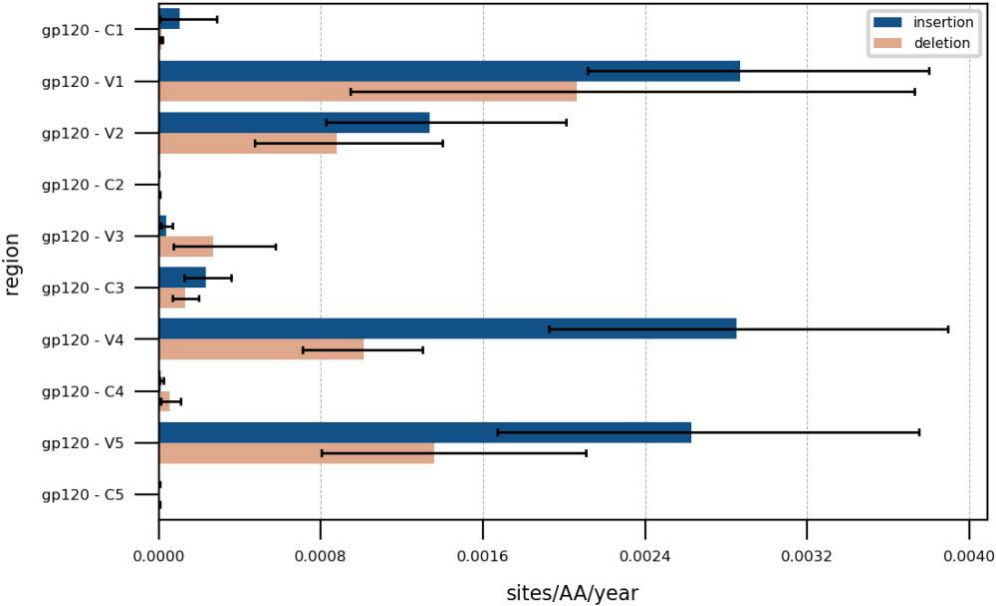
Rates comparison between variable (V1–V5) and conserved (C1–C5) regions of the gp120 glycoprotein.

In the subsequent analysis, we used the results from the IndelMaP–IndelMaP combination to examine various subsections of the *env* gene encoding the gp120 glycoprotein in greater detail. The inferred indel rates, as measured in events per amino acid per year, for the conserved (C1–C5) and variable (V1–V5) regions are shown in Fig. 5. The hypervariable loop V1 shows the highest rates with $2.87\times 10^{-3}$ and $2.06\times 10^{-3}$ for insertions and deletions respectively, then V2 with $1.34\times 10^{-3}$ and $8.76\times 10^{-4}$, V4 with $2.85\times 10^{-3}$ and $1.01\times 10^{-3}$, and V5 with $2.63\times 10^{-3}$ and $1.36\times 10^{-3}$. We found rates of insertions to be systematically higher than for deletions in these four regions, with an average of $2.42\times 10^{-3}$ versus $1.33\times 10^{-3}$ respectively. Finally, the C1, C2, C4, and C5 regions show near-null rates in comparison. The indel rates we report are approximately four times higher than those for HIV-1 subtype B by Palmer and Poon (Palmer and Poon 2019), but the overall pattern is similar, with V1 and V5 showing the highest rates, followed by V2 and V4, and V3 with the lowest. However, Palmer and Poon did not distinguish between insertions and deletions, and their method of tracking length differences in sequences with a binomial outcome does not account for overlapping or canceling indel events, limiting the relevance of this comparison. More broadly, we observed the highest rates for *env* with an average insertion rate higher than the deletion rate. The *gag*, *nef*, *vpr,* and *vpu* regions also demonstrate significantly higher rates compared with *pol*, *rev*, *tat*, and *vif* (supplementary fig. S19, Supplementary Material online).

**Fig. 6.**
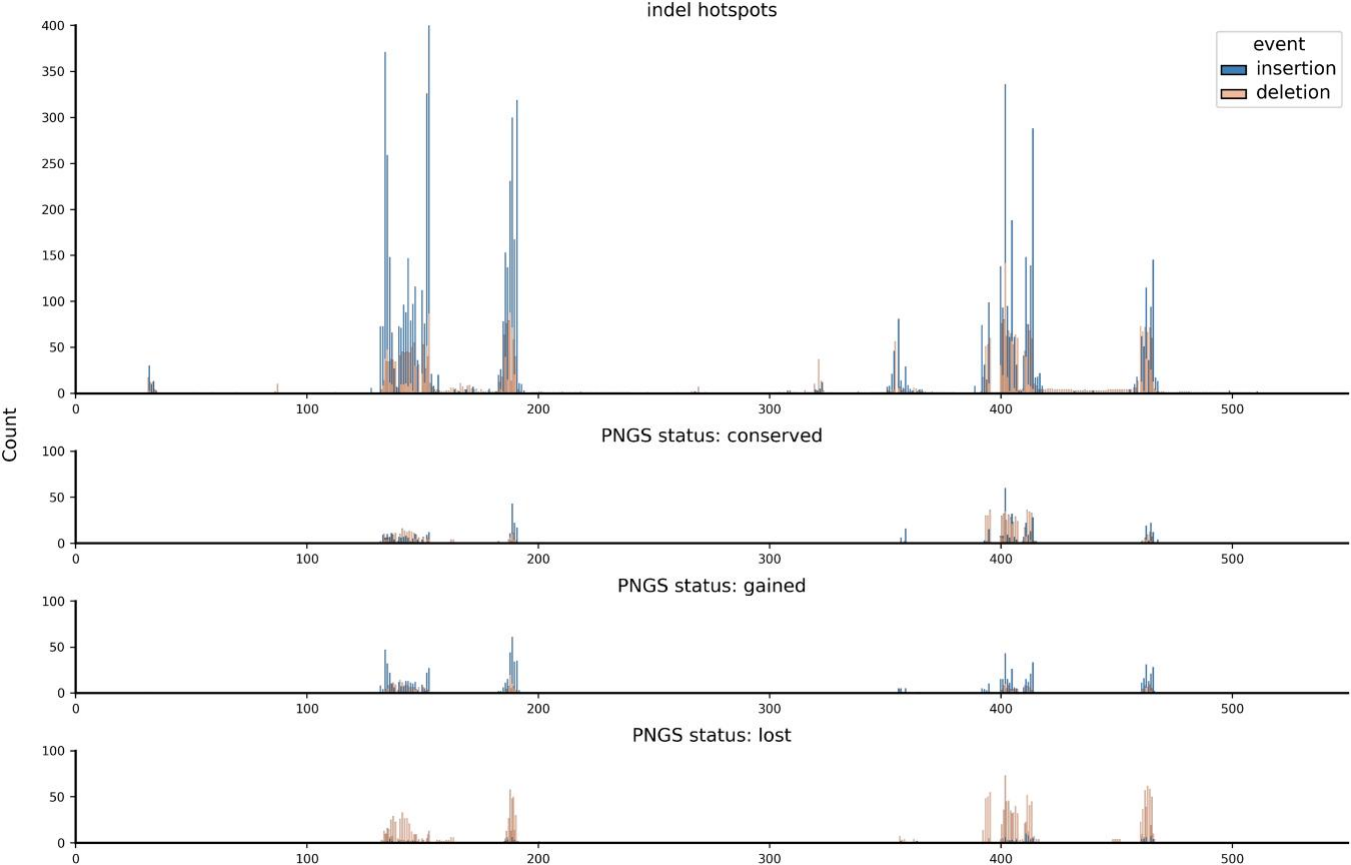
Conserved, gained, and lost potential N-linked glycosylation sites found on the gp120 glycoprotein compared to its indel hotspot profile.

In addition to calculating indel rates, our analysis specifically targeted potential N-linked glycosylation sites (PNGSs). These sites are crucial as mutations in PNGSs are known to impact viral infectivity and antibody response (Wang et al. 2013). To identify PNGSs, we utilized the regular expression “N[ˆP][ST][ˆP]”, where “[ˆP]” matches any amino acid except proline, and “[ST]” denotes either serine or threonine. Subsequently, we classified these sites based on whether they were introduced or lost due to indel events (Fig. 6). Conserved sites were those identified in both parent and child sequences. We observed significant hotspots spanning residues 132 to 153 (V1), 186 to 191 (V2), 400 to 415 (V4), and 460 to 467 (V5). Comparing these hotspot profiles to the overall indel hotspot profile, we note that while V1 is prominently featured in the overall indel hotspot profile, V4 and V5 exhibit substantially higher counts in the PNGS-targeted hotspot profiles.

## Discussion

Our comparative analysis of phylogenetic inference methods applied to the HIV-1 subtype B *env* gene provides valuable insights into the complex interplay between alignment and ASR methods, particularly regarding their sensitivity to insertions and deletions. We observe that hotspots for indel events, as revealed by the different methodologies evaluated for the entire env gene, display a consistent general pattern across methods. Further, although there are some differences, which we will discuss further below, indel events were globally inferred in the regions where they were expected according to the literature (Li et al. 1988; Wood et al. 2009). Indeed, we found significant hotspots to be successfully placed in the hypervariable loops V1, V2, V4, and V5 as well as in the conserved region C3, so named for structural reasons but also known to be relatively prone to evolutionary events (Hoffman et al. 2002).

Our main observation is the pronounced influence of alignment tools on the inferred indel patterns compared to the influence of ASR tools. While the plots showing hotspots over the entire *env* gene appear relatively similar due to the gene’s length overshadowing specific regions of interest, a closer analysis of the V1 hypervariable loop highlights the significant impact of the alignment tool in shaping the indel landscape, directly affecting the placement of indel events. The choice of alignment tool—MAFFT, ${\text{PRANK}}_{+F}$, IndelMaP, ProPIP, or Historian—leads to distinct indel hotspot and coldspot profiles. Specifically, MAFFT tends to imply a majority of deletions, regardless of the tool used for ancestral reconstruction. This pattern has been observed across the entire *env* region. This effect is attributable to MAFFT’s method of operation, which tends to overalign by forcing nonhomologous regions into alignment, does not differentiate between insertions and deletions, and treats all gap characters as deletions (Löytynoja and Goldman 2008). This leads to the production of artificially shorter alignments that lack consistency with the underlying phylogeny. We do not encounter this predominance of deletions when the MAFFT alignment is used by Historian, which provides an indel pattern that differs greatly from other tools using the same alignment. This is due to Historian iterating and refining the guide alignment. This particular situation will be dealt with further below. The count also appears higher for MAFFT alignments, which may be explained by the lack of inferred insertions at half positions and the accumulation of all indel events then considered as deletions and placed at full positions.

In contrast, the profiles obtained using ProPIP alignment show different characteristics, with indel events distributed more widely along the gene rather than being concentrated in specific hotspots, as seen with other methods. This pattern arises from ProPIP’s limitation to only model single-character indel events. As a consequence, it breaks long indels and spreads them more broadly. This effect is more visible in shorter regions (e.g. *rev*). For the same reason, ProPIP also demonstrates a higher indel event count than ${\text{PRANK}}_{+F}$ or IndelMaP.

In general, the indel patterns obtained are highly conserved between ancestral reconstruction methods when using the same alignment. This observation highlights that events placed during the alignment stage tend to be preserved in subsequent stages. However, reconstructions performed using Historian deviate from this pattern, offering profiles that differ significantly from those obtained by other tools using the same alignment. As Historian not only infers ancestors but, in the process, also locally refines the input MSA, it can rebalance the proportion between insertions and deletions when using the MAFFT alignment or override ProPIP’s tendency to distribute events more evenly. Notably, a cold spot appears between positions 145 and 149 of the V1 hypervariable region, inferred only when Historian is used for either alignment or reconstruction. While these observations confirm Historian’s capability to modify the input alignment, they do not contradict the above results or imply that the results are more accurate than other alternatives. The latter remark holds for all methods, as we lack ground truth and most observations here are phenomenological comparisons without knowledge about correctness.

The choice of tool for ASR shows a very limited impact on the result compared to the choice of alignment tool. Interestingly, when ASR is performed by GRASP or FastML using a MAFFT alignment, the rates between insertions and deletions are more balanced. This balance is explained by the fact that these two tools allow the reinsertion of residues, meaning a character can be inserted at a position where another had previously been deleted, thus not adhering to Dollo’s law (Farris 1977). The rates and lengths obtained for ${\text{PRANK}}_{+F}$ and IndelMaP alignments are highly similar, which aligns with the fact that both tools use the same algorithmic method for flagging indel events despite different approaches to alignment.

We also find that rates obtained with a ProPIP alignment, specifically with the ProPIP-ARPIP combination, tend to be higher, presumably due to the PIP model inferring only single residue events. This leads to a more fragmented distribution of indel events, resulting in inflated rates since they are calculated as events per amino acid per year. While this inflation is also observed when using ARPIP with other MSAs, the difference is not as pronounced as when using the ProPIP-ARPIP combination. The impact of the PIP model is also evident in the length profiles obtained with ProPIP alignment, where indels are significantly shorter. Interestingly, despite using the PIP model, ARPIP can retain information on longer indels when using MAFFT, ${\text{PRANK}}_{+F}$, IndelMaP, or Historian alignments (Jowkar et al. 2024). These observations also apply to the rates and lengths computed specifically for the V1 variable loop. Although we found the rates in this loop to be approximately three times higher than for the entire *env* gene, the effects of the various approaches discussed above are equally discernible, indicating that these effects are inherent to the methods and not dependent on the region studied (supplementary fig. S10, Supplementary Material online).

Similarly to hotspots, Historian can readjust rates and lengths despite biases in the alignment used. This is exemplified by the balanced rates between insertions and deletions observed with a MAFFT MSA or the reduced number of inferred single residues when using a ProPIP MSA compared to other tools on the same alignment. The local realignment leads to a mixture of the input MSA and Historian’s model. It is not clear which scenarios benefit from such a mixture. Thus, we recommend running Historian’s ASR in conjunction with its MSA for coherence.

Overall, we show that indel events placed during alignment are highly conserved during the ancestral reconstruction process. This observation underscores that the alignment step is a determining factor in discovering evolutionary dynamics and emphasizes the necessity of choosing a sequence alignment tool that is appropriate for the context of the study. MAFFT, for example, is an interesting aligner that offers the advantages of speed and applicability to datasets of weaker similarity (Katoh and Standley 2016) but appears poorly suited to the analysis of indel events, as shown by the disproportionately high deletion rates. As ProPIP only allows indels to be modeled as single residue events, it prevents the study of longer events that may occur. ${\text{PRANK}}_{+F}$, IndelMAP, and Historian show very similar results in terms of rates and lengths despite a different hotspot profile for Historian, and all appear suitable for such an analysis. As mentioned above, it’s important to note that we have no way of knowing the true placement of indels. A comparison of these different methods has already been conducted using simulated data to determine the actual positions of such events within the tree (Iglhaut et al. 2024). However, using a model to simulate molecular evolution naturally introduces bias into the data. Since the lack of ground truth is an intrinsic challenge in the field, we have chosen to work exclusively with nonsimulated data in this study to investigate the observable differences in real data. Simulation studies show that different MSA tools carry relative biases. For example, previous work has shown that MAFFT often produces shorter MSAs compared to PRANK (Löytynoja and Goldman 2008), a trend observed in our data as well. However, as mentioned above, absolute biases cannot be directly quantified in our dataset. Further characterization of MSAs, such as indel length distributions, gap/indel rates, and MSA lengths, provides useful insights into their impact on ASR estimates (Jowkar et al. 2024). While absolute biases remain difficult to measure, studies like those from PRANK (Löytynoja and Goldman 2008) and IndelMaP (Iglhaut et al. 2024), which explore MSA tool comparisons in simulated data, offer valuable references.

We note that ${\text{PRANK}}_{+F}$ has considerable limitations when working with large quantities of input data and therefore requires subsetting (i.e. for *env*, we had to work on subsets of 200 sequences of length 850 approximately, which was not necessary with IndelMAP or Historian). While the choice of ASR tool has a smaller impact on indel pattern results, some considerations remain significant. GRASP and FastML allow characters to be reinserted, which contradicts the principle that inserted characters cannot be ancestral to each other. Additionally, GRASP is restricted to protein sequences. Both FastML and ARPIP require subsetting on larger datasets. Historian, even when instructed to maintain the input MSA unchanged, uses the alignment merely as a guide if it does not fit its underlying model. This approach may necessitate realignment, posing challenges, especially if we want the ancestral reconstruction to align with a carefully curated MSA. We acknowledge that the relationship between MSA and ASR is asymmetrical, with MSA having a larger impact not because it is more fundamental than ASR, but because the MSA step precedes ASR in the analysis. The inferred MSA enforces character homologies which effectively constrains the subsequent ASR step, making the choice of MSA tool crucial. Although Historian refines MSA iteratively, it still follows the classical MSA-first, ASR-next workflow. Ideally, MSA and ASR would be performed simultaneously in a joint inference framework under a proper model, but this remains a challenging task due to the circularity issue present in many phylogenetic and MSA-related analyses. Thus, while MSA tools influence ASR results, it is not that MSA is inherently more fundamental than ASR, but that the order of operations introduces a dependency that must be considered in these analyses.

The subsequent analysis of indel rates and the impact of indels on PNGS demonstrates that indel-aware methods can be effectively leveraged to study indel patterns in specific regions of interest and functional sites. While we chose the IndelMaP–IndelMaP combination for this study, other combinations, such as Historian–Historian or PRANK–IndelMaP, could also be used. Given the larger dataset, the first two combinations are optimal as they do not require subsetting. The results show that indel-aware methods can provide valuable insights into indel patterns. These findings suggest that indel events contribute to the gain and loss of PNGS, particularly in V4 and V5. This insight could direct future studies, emphasizing the importance of considering indel patterns in the context of viral evolution and immune evasion mechanisms. Finally, our findings reveal that adopting an indel-aware approach enables the distinction between insertion and deletion events. This separation provides new insights into their individual roles and contributions to the process of evolutionary dynamics.

## Material and Methods

**Fig. 7.**
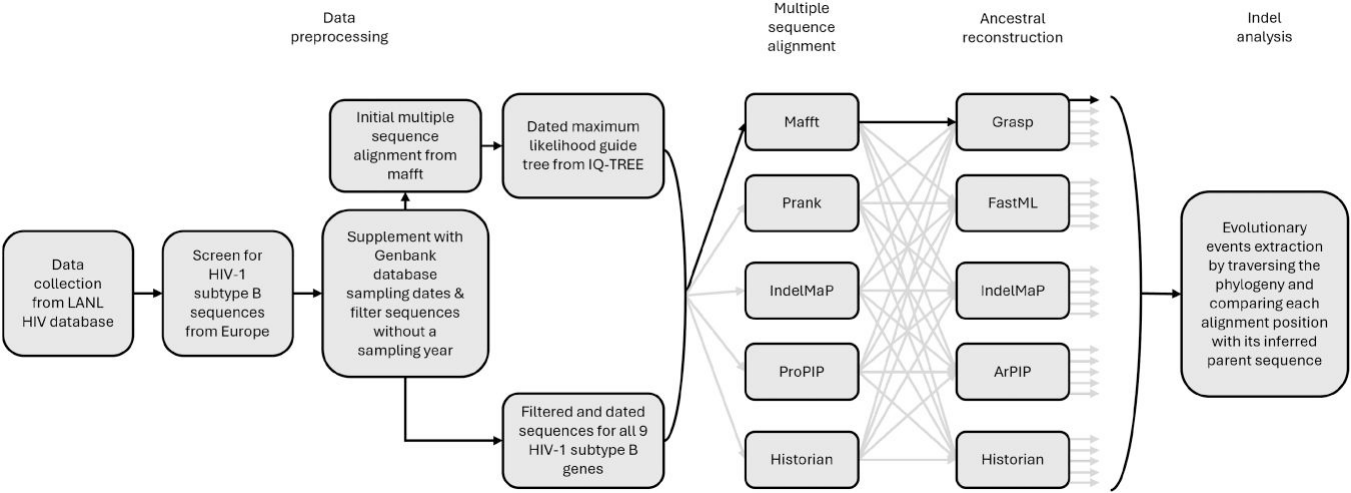
Method flowchart for data sourcing, ancestral sequence reconstruction, and indel analysis.

We analyzed five tools for alignment (MAFFT, ${\text{PRANK}}_{+F}$, IndelMaP, ProPIP, and Historian) and five for ancestral sequence reconstruction (GRASP, FastML, IndelMaP, ARPIP, and Historian). Each combination of MSA and ASR tool was run on a set of genomic regions. Based on the resulting MSAs and phylogenies labeled with ancestral sequences, we inferred insertions and deletion events and their rates, lengths, and positions in the sequences studied (Fig. 7). Indel analysis was also conducted on *env* subregions specifically.

### Data Collection and Preprocessing

We first collected amino acid sequences of all nine HIV-1 subtype B genes from the Los Alamos National Laboratory HIV database (http://www.hiv.lanl.gov, accessed 2023 Aug 23), extracted sequences originating from Europe and subsequently supplemented them with a more detailed sampling date from the Genbank database (https://www.ncbi.nlm.nih.gov/genbank/, accessed 2023 Aug 23). This was done by querying accession numbers, and only sequences with at least a known sampling year were retained for further analysis. Sequences selected for analysis consisted of 781, 647, 485, 495, 488, 468, 651, 698, and 544 for *env*, *gag*, *nef*, *pol*, *rev*, *tat*, *vif*, *vpr*, and *vpu*, respectively, with sampling years ranging from 1983 to 2021.

### Phylogenetic Inference

To evaluate the method’s impact on the observed indel pattern, we inferred sequence alignments and ancestral sequences using the tools mentioned above. This resulted in a total of 25 possible combinations. For each gene, we first inferred an initial alignment using MAFFT (Katoh et al. 2002) and used it to estimate and date a guide maximum likelihood phylogeny with IQ-TREE (Nguyen et al. 2015; To et al. 2016), as this tool treats gaps as missing data, minimizing the impact of alignment gaps on tree inference. We then outgroup rooted the resulting phylogeny with the K03455 reference sequence and used it as a guide tree for all multiple sequence alignments and ancestral reconstructions. This implies that indels are inferred relative to K03455, rather than to a true ancestral sequence—an important distinction given that K03455 is itself a subtype B genome. However, K03455 is widely used as the primary reference genome for HIV-1 at the Los Alamos HIV Databases (https://www.hiv.lanl.gov/content/sequence/HIV/MAP/landmark.html, accessed 2025 Jan 23), making it a practical and consistent choice for comparative analyses within subtype B. We conducted multiple sequence alignments of HIV-1 subtype B sequences using MAFFT (Katoh et al. 2002), IndelMaP (available at https://github.com/acgteam/indelMaP), ${\text{PRANK}}_{+F}$ (Löytynoja and Goldman 2008), Historian (Holmes 2017) and ProPIP (Maiolo et al. 2021). Prior to alignment with MAFFT, the phylogenetic tree underwent modification as we replaced identifiers by incrementing integers while maintaining their order and converted it from newick to a MAFFT-compatible format using a Ruby script made available in the MAFFT documentation. We also rewrote the input tree for IndelMaP using decimal instead of scientific notation. Finally, we created subsets for ProPIP as the computational complexity of its progressive algorithm did not allow such a large number of sequences to be processed. We split the sequences into three subsets for *env*, *gag*, *vif*, and *vpr*, and into two subsets for all remaining genes. To preserve as much of the original phylogeny as possible, we performed subsetting by traversing postorder-wise the trees while splitting leaves into groups. Subsequently, we pruned the tree for each subgroup and extracted all corresponding sequences into a new file. We then added the reference sequence K03455 to both the tree and the Fasta if it was not already present and set it as an outgroup. We also used the same subsets of the *env* gene for the ${\text{PRANK}}_{+F}$ alignment, as it failed to run on a large number of sequences.

MSA with MAFFT (v7.505) utilized the auto option, selecting an appropriate algorithm according to data size and the HIV between-host amino acid substitution frequencies matrix (Nickle et al. 2007). IndelMaP (build from https://github.com/acg-team/indelMaP) employed the same HIVb model. ${\text{PRANK}}_{+F}$ (v.170427) utilized the WAG protein substitution matrix (Whelan and Goldman 2001) as well as the +F option to make inferred insertions permanent and was run once. ProPIP (build from https://github.com/acg-team/ProPIP) used the WAG matrix, the PIP meta model, the ram dynamic programming alignment method, and a constant rate distribution without optimization. Historian (v1.1.0) was run on default using the WAG matrix. To ensure consistency across our analyses, all alignment software were used without specific tuning to the datasets.

We then used the resulting MSAs as input alongside the guide tree to reconstruct ancestral sequences, applying GRASP (Ross et al. 2022), FastML (Ashkenazy et al. 2012), IndelMaP, Historian, and ARPIP (Jowkar et al. 2023) to each previously obtained alignment. MSA processing involved removing ambiguous characters from the GRASP and ARPIP input sequences as they cannot handle such characters by randomly replacing X with one of the 20 standard amino acids, B with D or N, Z with E or Q, and J with I or L. For the FastML input, we rewrote all phylogenies, setting the minimum branch length value to $10^{-6}$ expected substitutions per site to avoid excessively low values, as FastML failed to run with overly short branch length. Finally, subsetting was also carried out for FastML and ARPIP, following the method presented above. For FastML, all MSAs not derived from ProPIP or the *env* alignment by ${\text{PRANK}}_{+F}$ were split four times. For ARPIP, alignments were split six times for *env*, *gag*, and *nef*, and four times for all other genes. GRASP (version 04-May-2023) utilized the JTT protein model (Jones et al. 1992). FastML (v3.11) used the WAG substitution matrix with no branch length optimization, using both maximum likelihood and parsimony for indel reconstruction and no gamma distribution. IndelMaP (build on https://github.com/acg-team/indelMaP, Rust implementation) was run with the HIV between-host protein matrix and Historian with the default parameter and the WAG matrix. Finally, ARPIP (build on https://github.com/acg-team/bpp-arpip) used the WAG matrix as well, and the PIP meta model, with no previous estimates of evolutionary parameters lambda and mu.

### Indel Pattern Inference

IndelMaP returns indel information in a Fasta file containing the alignment of all sequences (leaves and internal nodes), differentiating between gap characters “-” indicating a residue deleted earlier in the phylogeny, and placeholders “*” indicating a residue inserted later at that position. The inserted residue is indicated by a lowercase letter. We used this output format as is for further analysis and converted outputs from other phylogenetic tools to the same format. Alongside the aligned ancestral sequences, ARPIP returns a list of all indel events and their positions in the phylogenetic tree and alignment. We reconstructed an MSA from internal and external nodes similar to that of IndelMaP by traversing the tree in a preordered way for each position in the alignment and using the adapted character “-”, “*”, upper or lower case according to the list of indel events provided by ARPIP. GRASP, FastML, and Historian provide no indel information but only the reconstructed and aligned ancestral sequences. We estimated indel points by traversing the tree in preorder for each position in the alignment and comparing the residue at that position with that at the same position in the parent sequence. As long as no residue was found, we used a placeholder character (“*”); when a residue appeared, we considered it an insertion and noted it with a lowercase letter, and if a residue disappeared, the gap character “-” was used for all descendant sequences not containing a residue at that site. A multiple sequence alignment containing leaves, internal nodes, and indel events alongside the corresponding phylogenetic tree was thus obtained for each tool combination and later used for indel analysis.

### Indels Analysis

Once a complete phylogeny including ancestral sequences with distinguished gap and placeholder characters had been obtained for each combination and genome region, we traversed the phylogeny in preorder and compared each alignment position with its parent sequence. This allowed us to extract a list of all evolutionary events, containing identifiers of parent and child sequences, the time to the most recent common ancestor (tMCRA) in years, the type of event (insertion or deletion), the amino acid involved, and the concerned genome region along with its length. We also collected the position of the residue relative to the alignment, with and without placeholder characters, and relative to the reference sequence K03455. We saved inserted sites, which inherently are not present in the reference sequence, using half positions, i.e. if an insertion is located between positions two and three, the event’s position relative to the reference is 2.5. As tools following Dollo’s law (i.e. IndelMaP) do not allow a deleted residue to reappear, they result in a maximal count of one insertion at integer positions. To get the tMRCA in years, we divided the value of the corresponding branch length in expected number of substitutions per site by the substitution rate computed by IQ-TREE relative to each gene. The Wasserstein distance between inferred indel hotspot distributions was computed using the unweighted wasserstein_distance function from the scipy.stats. python library (Virtanen et al. 2020).

We also screened parent and child sequences around each event using the regular expression “N[ˆP][ST][ˆP]” to find potential N-linked glycosylation sites (PNGSs) and determine whether any had appeared or disappeared as a result of an evolutionary event. The expression “[ˆP]” would match any amino acid except proline and “[ST]” either serine or threonine.

By iterating this list, we computed the length of each indel by considering multiple residues inserted or deleted along the same branch as part of a single indel event, even if placeholders or preexisting gaps were present from other branches in the alignment. We considered the residues to be part of a continuous evolutionary event along that specific branch. The length of this indel is then the total number of residues inserted or removed as part of this single, continuous evolutionary event on the branch. We computed rates by considering each child node in the event list and counting the number of events, respectively insertion, deletion, or substitution occurring there, for a given region. This event count was then divided by the number of residues in the relevant region of the parent sequence and by the tMRCA in years. We ignored branches shorter than 0.001 years to avoid over-inflating rates due to division by an inappropriately low value, especially when the concerned region was also short.

## Supplementary Material

evaf119_Supplementary_Data

## Data Availability

Sequence data and scripts underlying this article are available at https://github.com/acg-team/Indel-Pattern-Analysis-HIV.
