# Supplementary material for: Impact of Phylogenetic Method Choice on Indel Analyses in HIV-1 Subtype B
Source: Genome Biol Evol. 2025 Jun 12;17(6):evaf119. doi: 10.1093/gbe/evaf119 (PMC12188288; doi:10.1093/gbe/evaf119)
Supplement: evaf119_Supplementary_Data [file evaf119_supplementary_data.pdf]

Supplementary Material

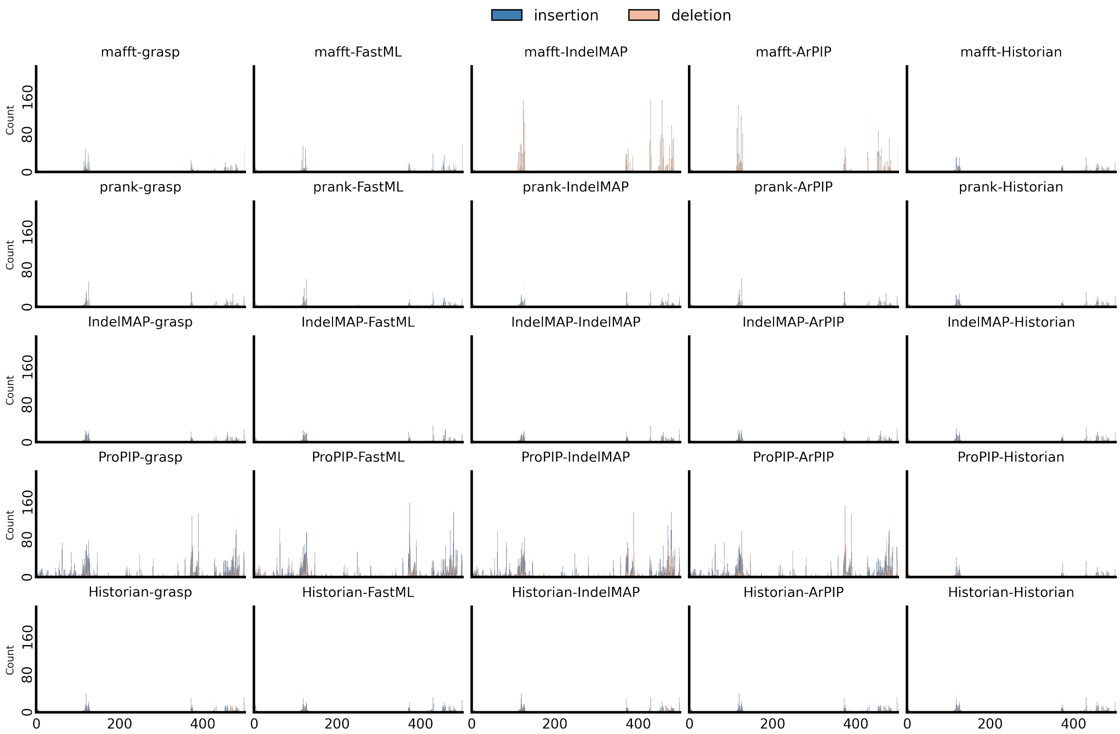

Figure S1: Insertion and deletion hotspots on the full *gag* gene according to the various phylogenetic methods used. Abscissa shows the position of the event on the reference sequence K03455 and ordinate shows the count of events.

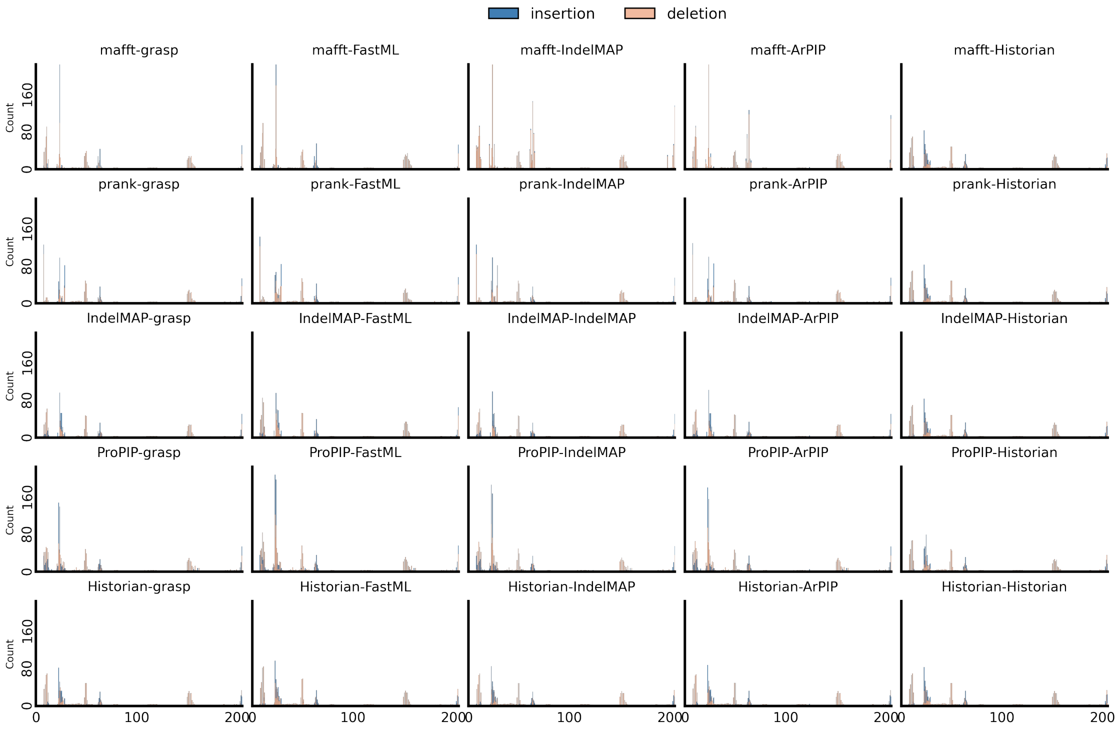

Figure S2: Insertion and deletion hotspots on the full *nef* gene according to the various phylogenetic methods used. Abscissa shows the position of the event on the reference sequence K03455 and ordinate shows the count of events.

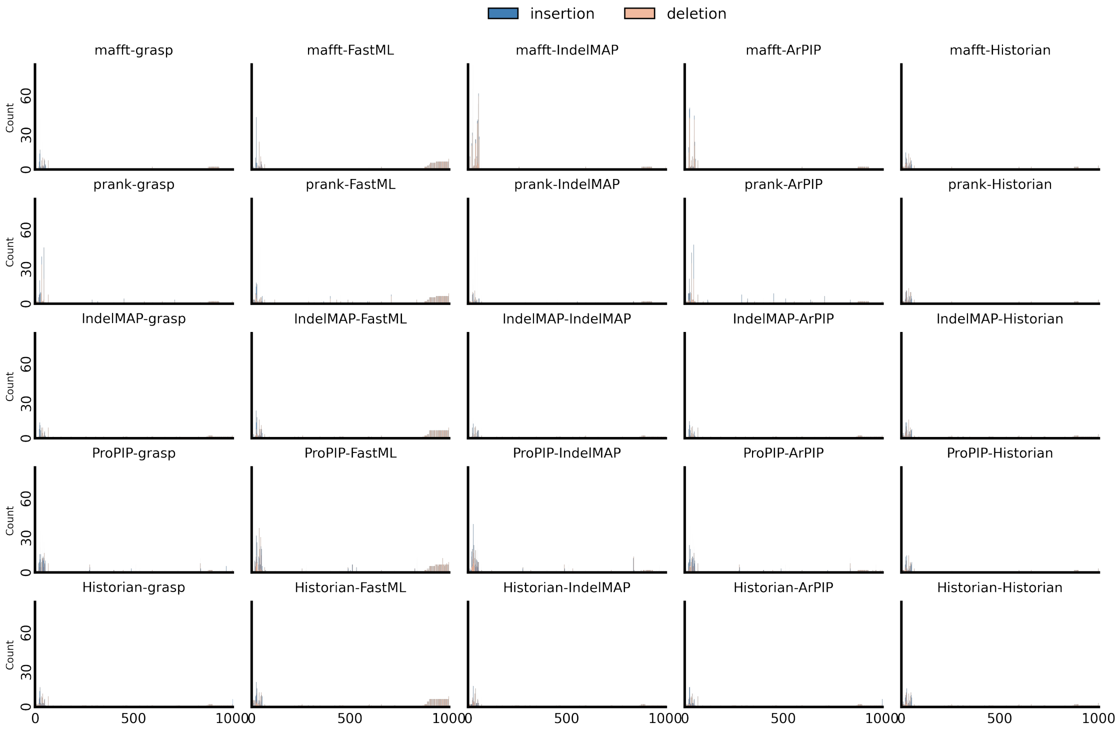

Figure S3: Insertion and deletion hotspots on the full *pol* gene according to the various phylogenetic methods used. Abscissa shows the position of the event on the reference sequence K03455 and ordinate shows the count of events.

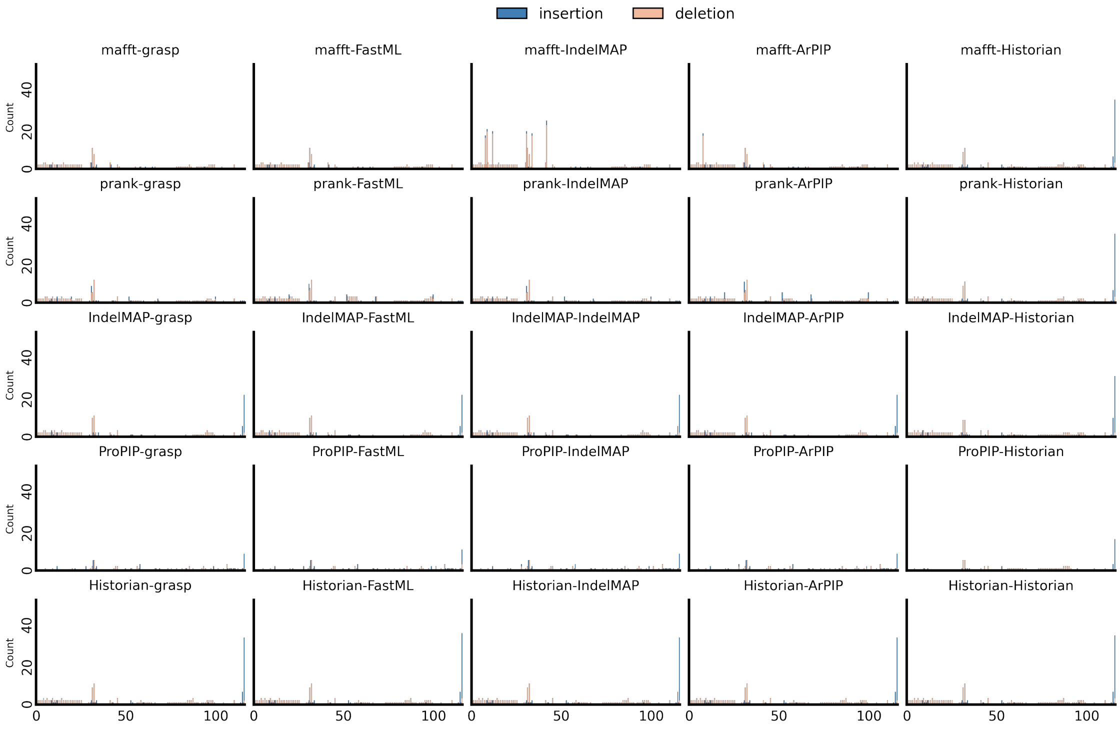

Figure S4: Insertion and deletion hotspots on the full *rev* gene according to the various phylogenetic methods used. Abscissa shows the position of the event on the reference sequence K03455 and ordinate shows the count of events.

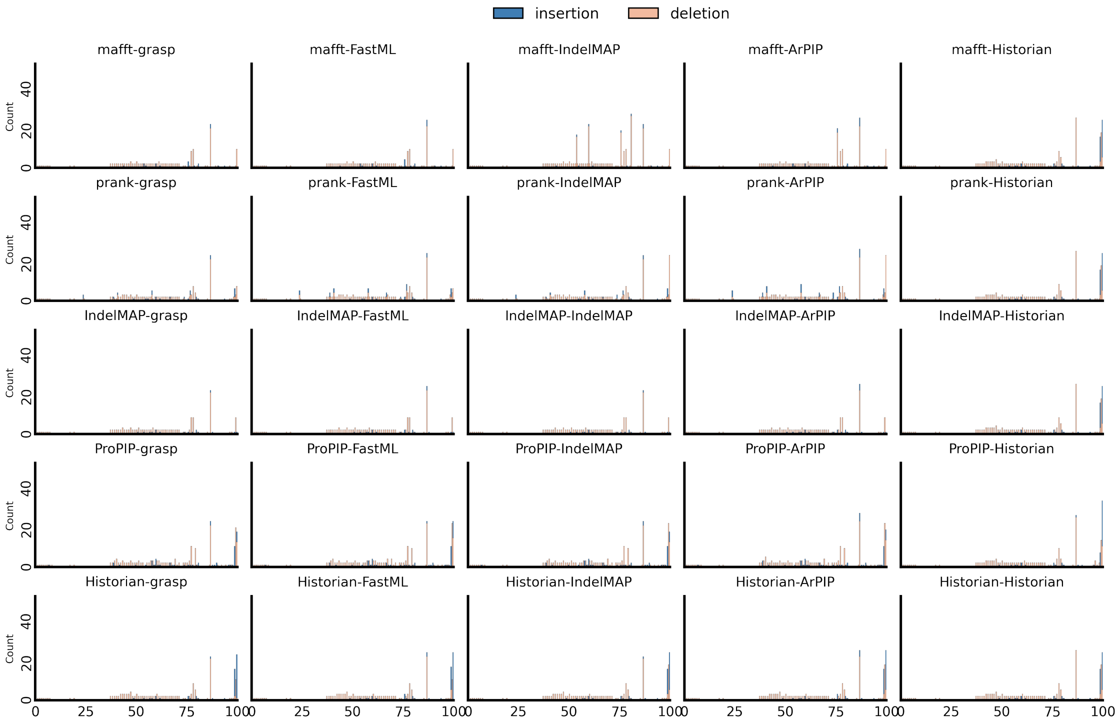

Figure S5: Insertion and deletion hotspots on the full *tat* gene according to the various phylogenetic methods used. Abscissa shows the position of the event on the reference sequence K03455 and ordinate shows the count of events.

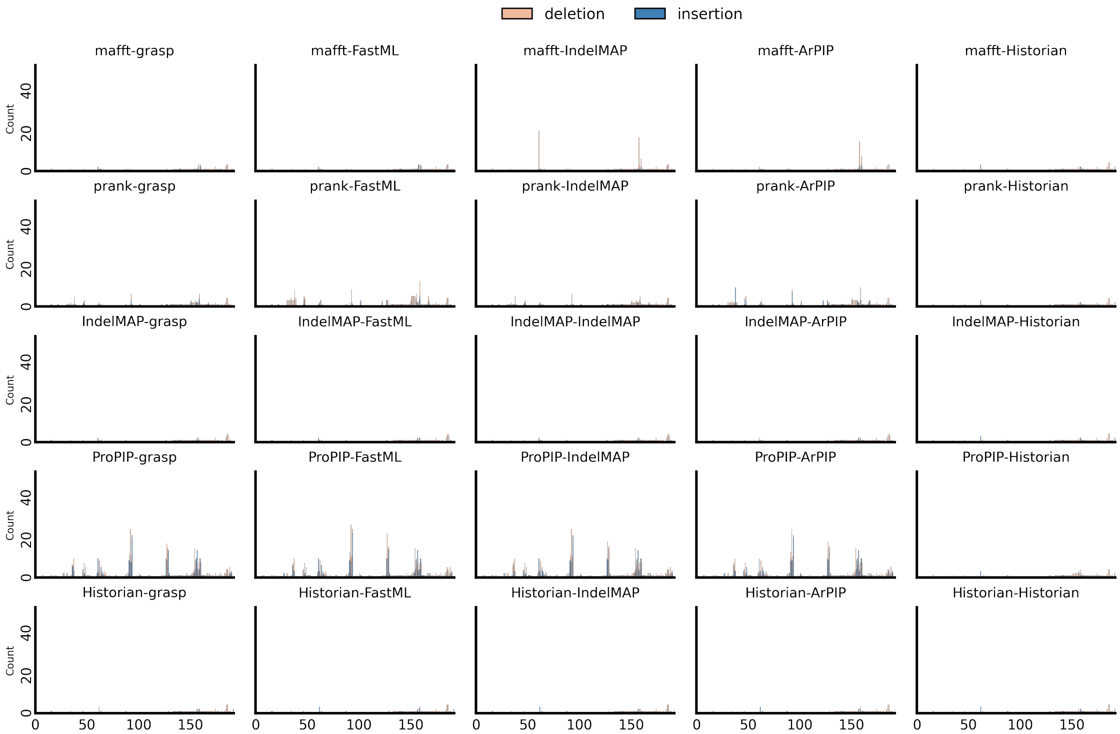

Figure S6: Insertion and deletion hotspots on the full *vif* gene according to the various phylogenetic methods used. Abscissa shows the position of the event on the reference sequence K03455 and ordinate shows the count of events.

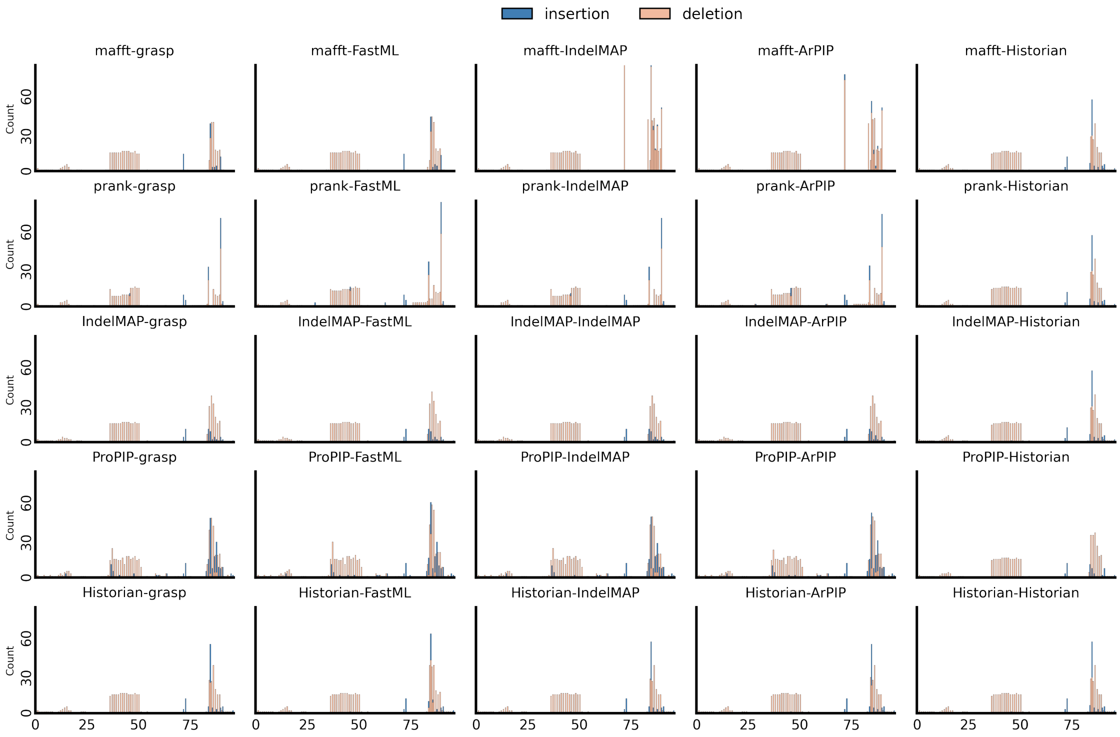

Figure S7: Insertion and deletion hotspots on the full *vpr* gene according to the various phylogenetic methods used. Abscissa shows the position of the event on the reference sequence K03455 and ordinate shows the count of events.

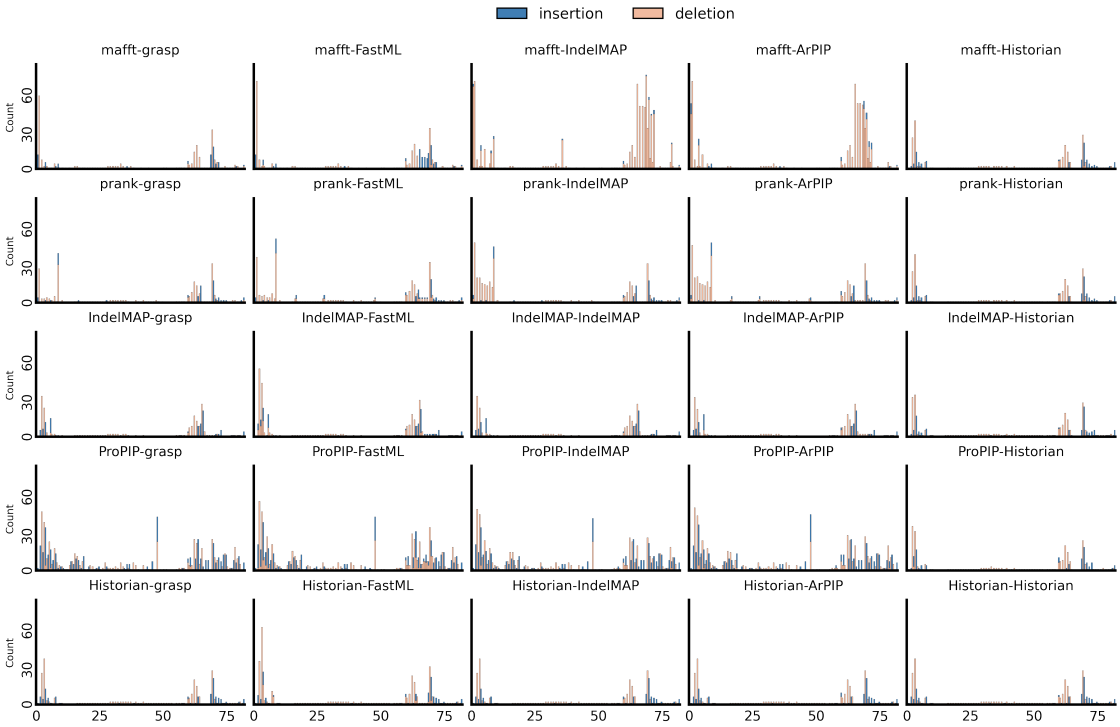

Figure S8: Insertion and deletion hotspots on the full *vpu* gene according to the various phylogenetic methods used. Abscissa shows the position of the event on the reference sequence K03455 and ordinate shows the count of events.

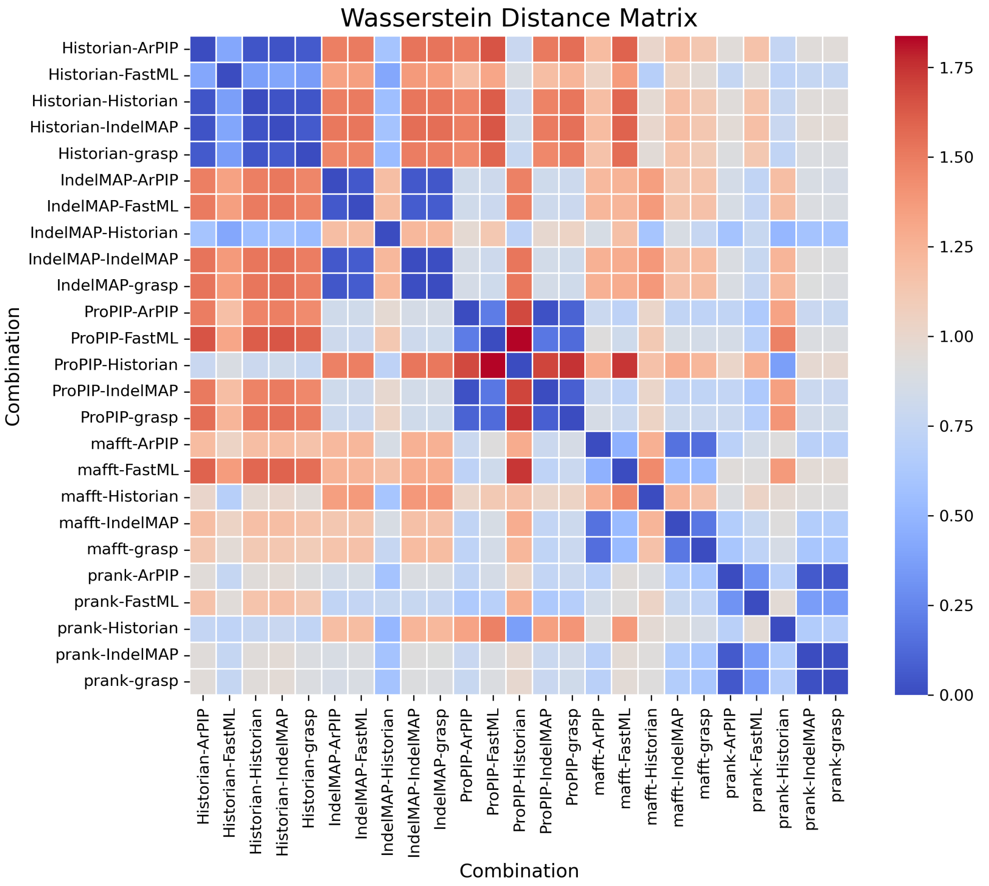

Figure S9: Wasserstein distance matrix between all method combinations. Wasserstein distances where computed between indel distribution inferred by the different methods on the V1 hypervariable loop of the *env* gene.

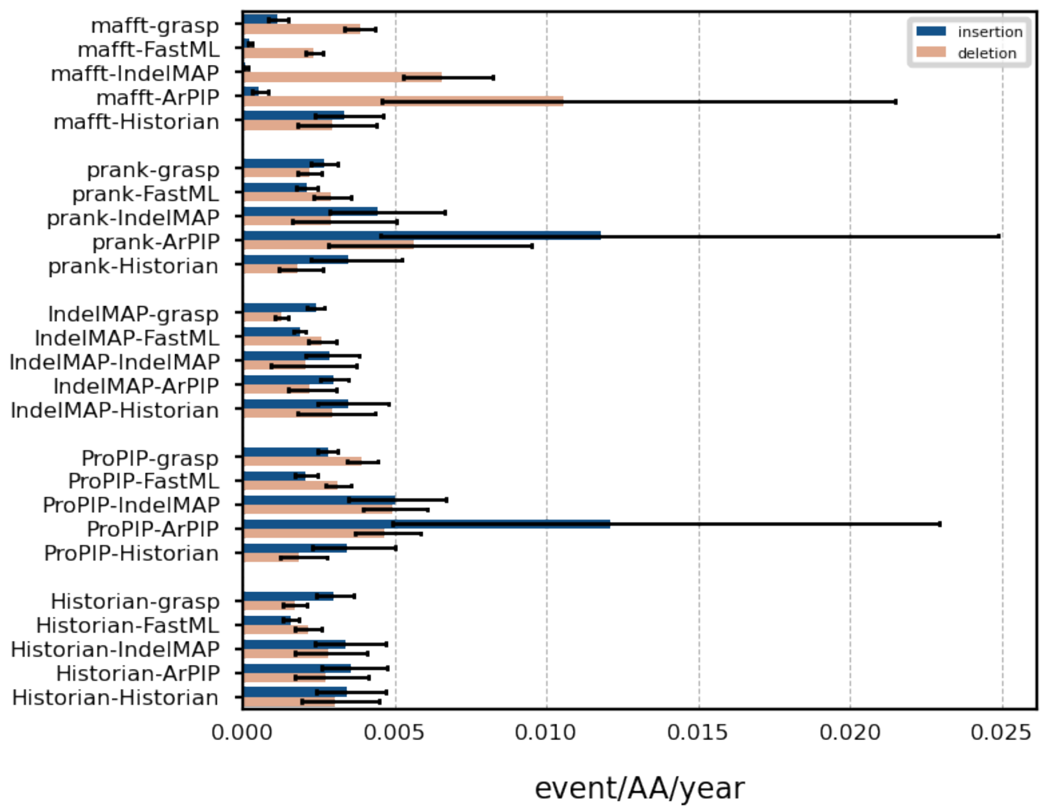

Figure S10: Indel rates comparison between the various phylogenetic methods used for the hypervariable loop V1 of the *env* gene.

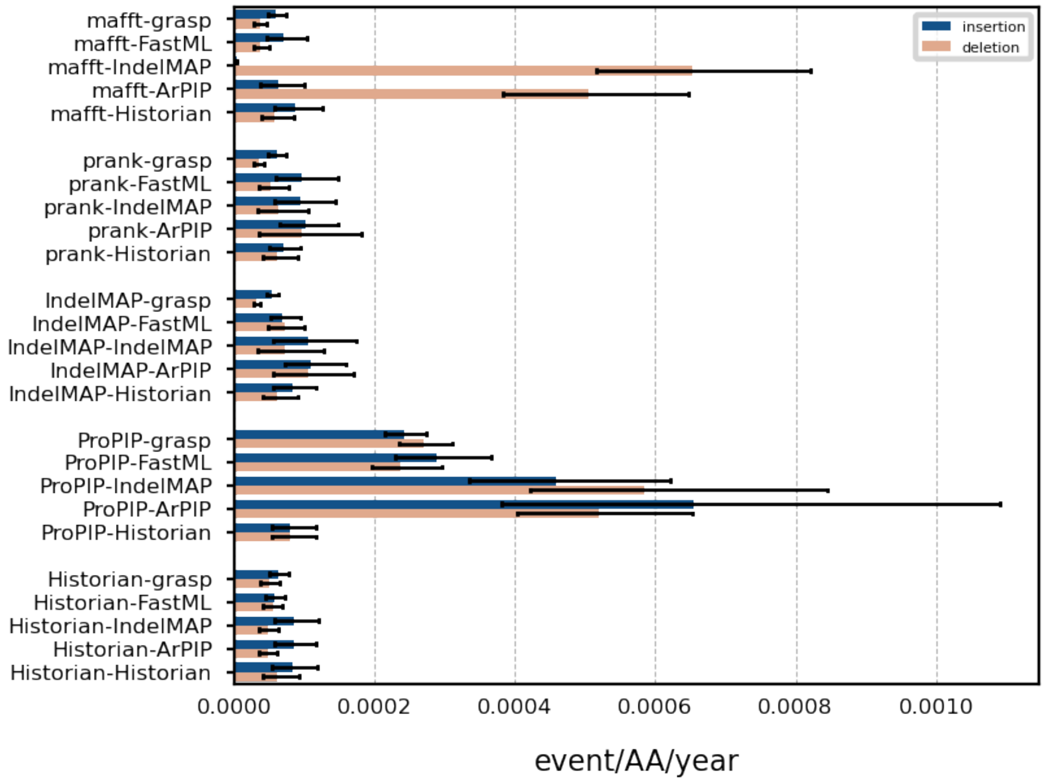

Figure S11: Indel rates comparison between the various phylogenetic methods used for the full *gag* gene.

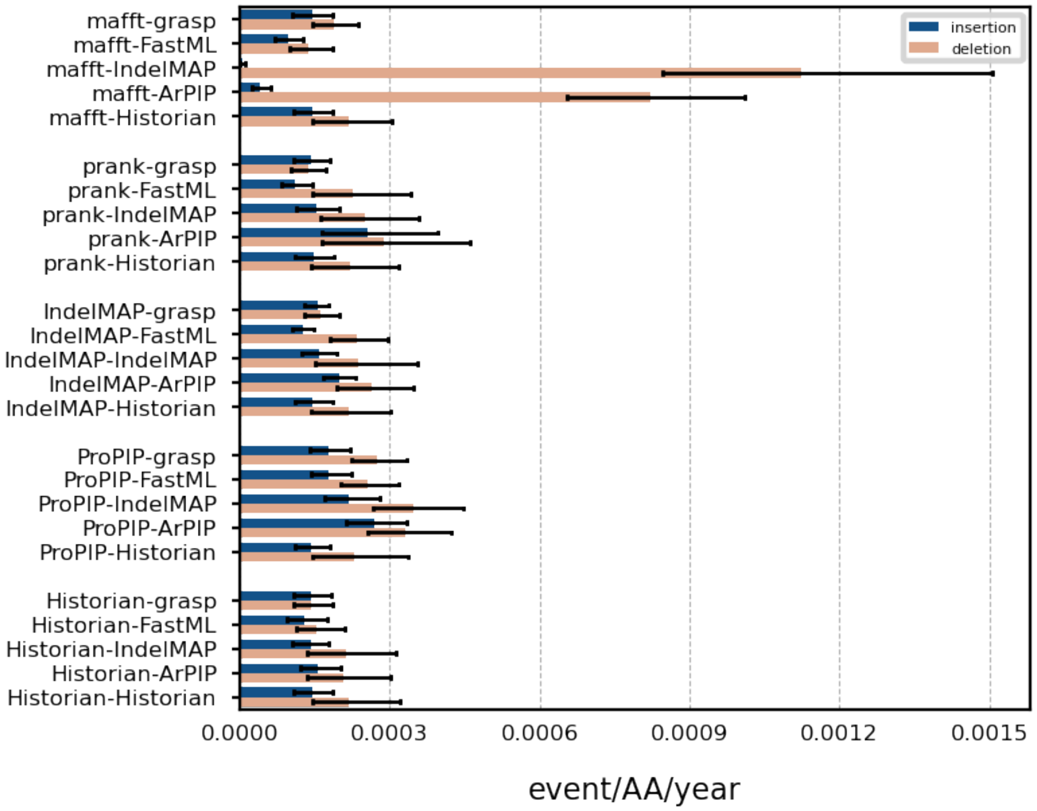

Figure S12: Indel rates comparison between the various phylogenetic methods used for the full *nef* gene.

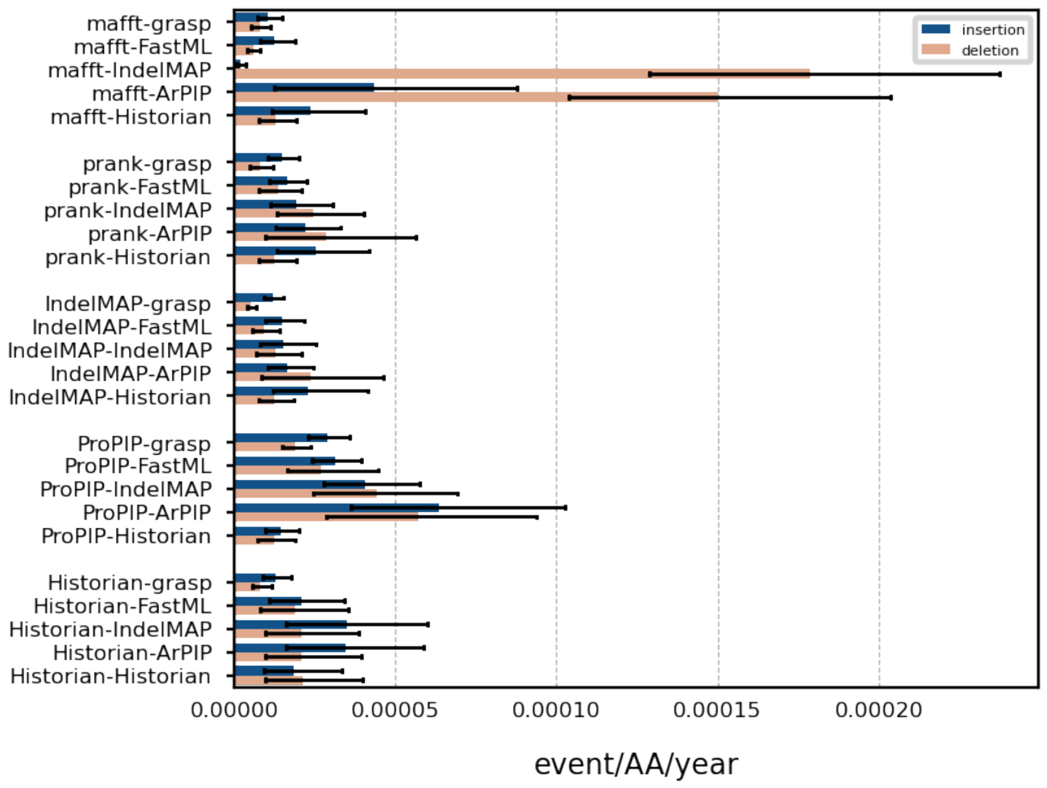

Figure S13: Indel rates comparison between the various phylogenetic methods used for the full *pol* gene.

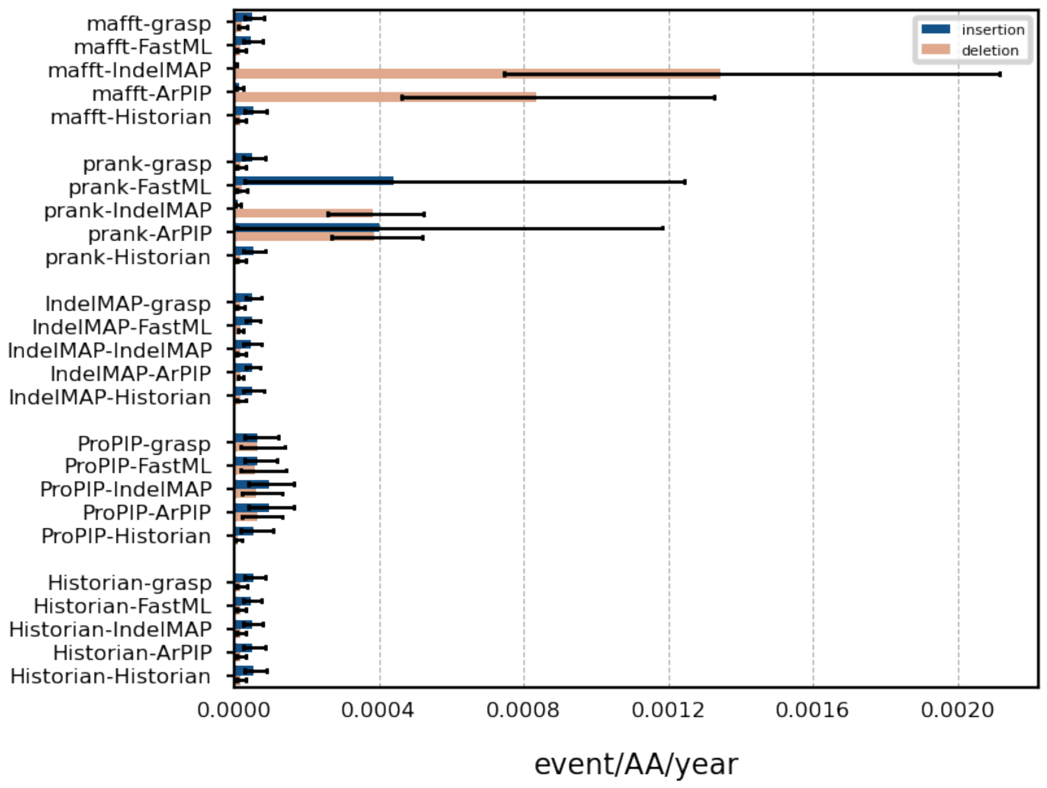

Figure S14: Indel rates comparison between the various phylogenetic methods used for the full *rev* gene.

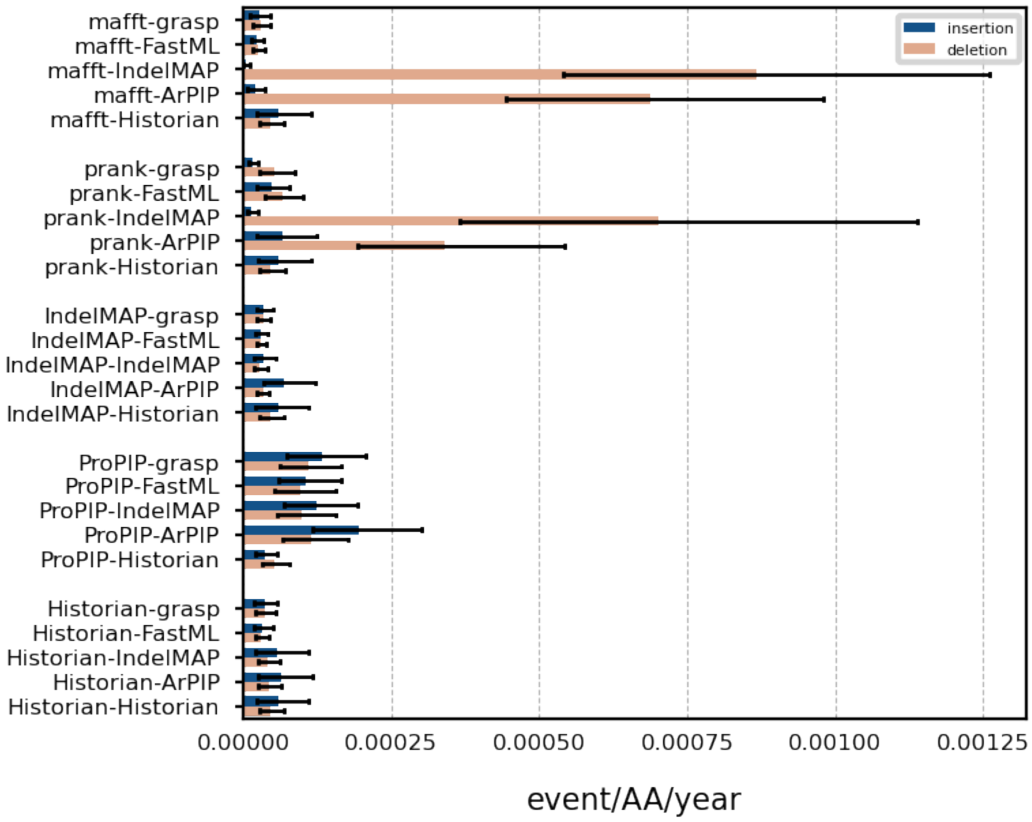

Figure S15: Indel rates comparison between the various phylogenetic methods used for the full *tat* gene.

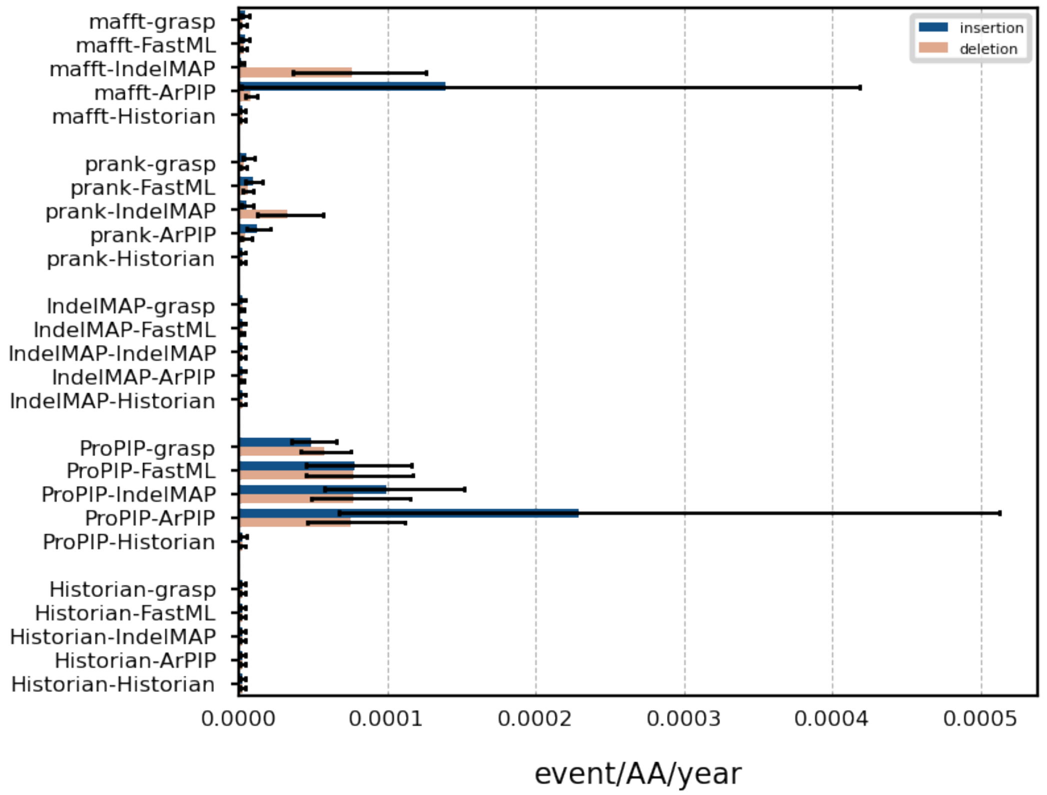

Figure S16: Indel rates comparison between the various phylogenetic methods used for the full *vif* gene.

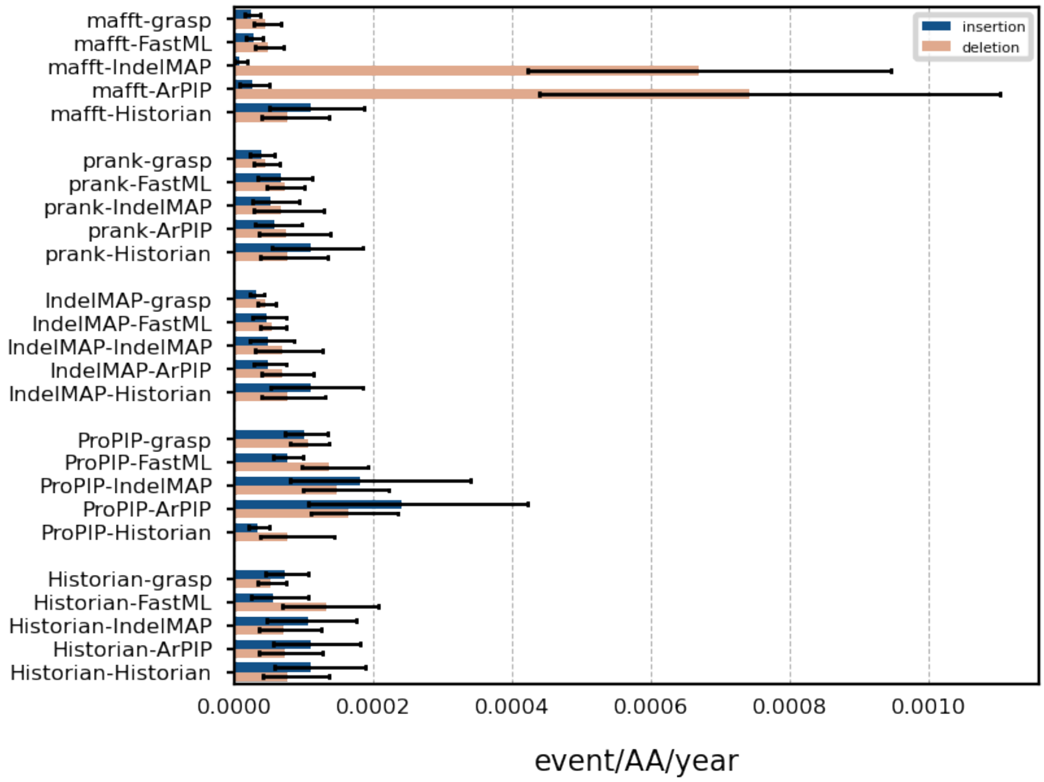

Figure S17: Indel rates comparison between the various phylogenetic methods used for the full *vpr* gene.

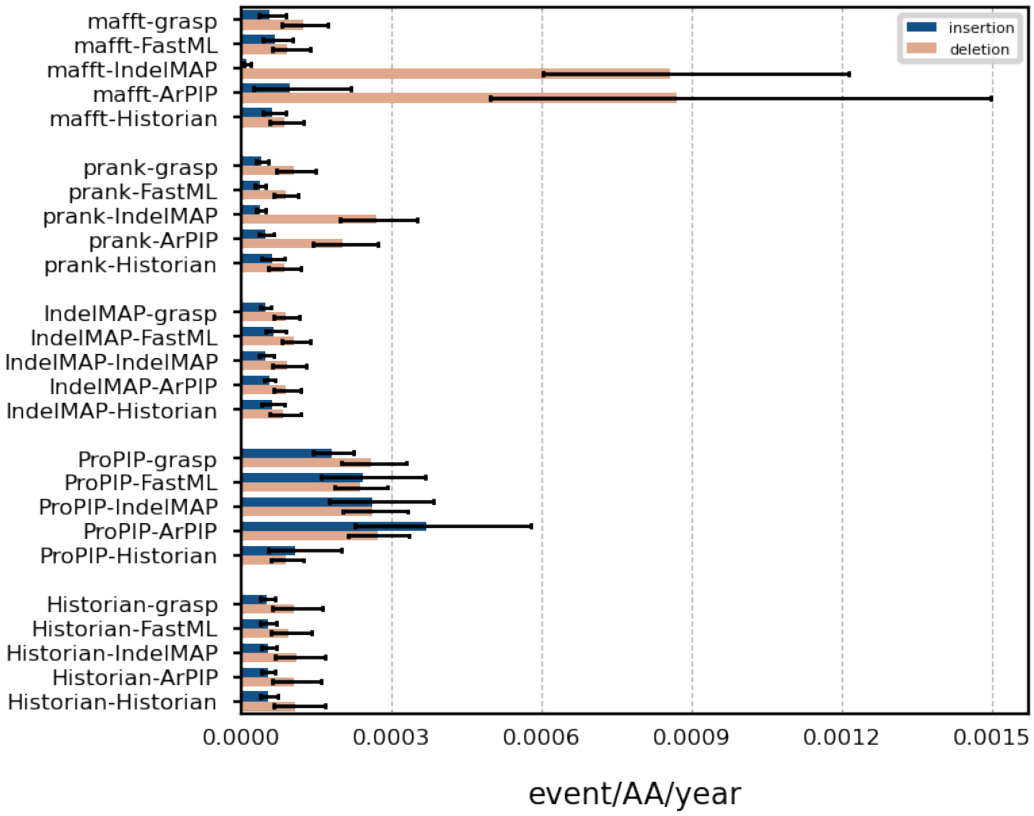

Figure S18: Indel rates comparison between the various phylogenetic methods used for the full *vpu* gene.

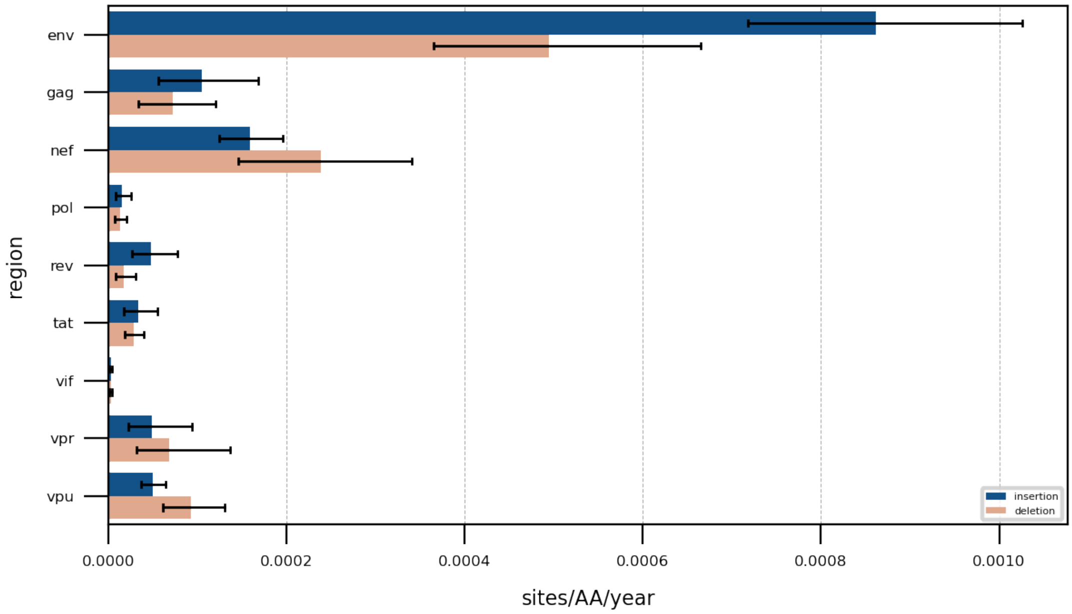

Figure S19: Comparison of indel rates inferred using IndelMaP-IndelMaP between the nine HIV-1 subtype B genes.
